# Design of discotic liquid crystal enabling complete switching along with memory of homeotropic and homogeneous alignment over a large area†

**DOI:** 10.1039/d2sc03677k

**Published:** 2022-07-19

**Authors:** Yoshiaki Shoji, Miki Kobayashi, Atsuko Kosaka, Rie Haruki, Reiji Kumai, Shin-ichi Adachi, Takashi Kajitani, Takanori Fukushima

**Affiliations:** Laboratory for Chemistry and Life Science, Institute of Innovative Research, Tokyo Institute of Technology 4259 Nagatsuta, Midori-ku Yokohama 226-8503 Japan fukushima@res.titech.ac.jp; Department of Chemical Science and Engineering, School of Materials and Chemical Technology, Tokyo Institute of Technology 4259 Nagatsuta, Midori-ku Yokohama 226-8503 Japan; Photon Factory, Institute of Materials Structure Science, High Energy Accelerator Research Organization 1-1 Oho Tsukuba 305-0801 Japan; Open Facility Development Office, Open Facility Center, Tokyo Institute of Technology 4259 Nagatsuta, Midori-ku Yokohama 226-8503 Japan kajitani.t.ab@m.titech.ac.jp; RIKEN SPring-8 Center 1-1-1 Kouto, Sayo Hyogo 679-5148 Japan

## Abstract

The alignment control of discotic columnar liquid crystals (LCs), featuring a low motility of the constituent molecules and thus having a large viscosity, is a challenging task. Here we show that triphenylene hexacarboxylic ester, when functionalized with hybrid side chains consisting of alkyl and perfluoroalkyl groups in an appropriate ratio, gives a hexagonal columnar (Col_h_) LC capable of selectively forming large-area uniform homeotropic or homogeneous alignments, upon cooling from its isotropic melt or upon application of a shear force at its LC temperature, respectively. In addition to the alignment switching ability, each alignment state remains persistent unless the LC is heated to its melting temperature. *In situ* X-ray diffraction analysis under the application of a shear force, together with polarized optical microscopy observations, revealed how the columnar assembly is changed during the alignment-switching process. The remarkable behavior of the discotic LC is discussed in terms of its rheological properties.

## Abstract 2

A columnar liquid crystal consisting of a triphenylene hexacarboxylic ester mesogen and semifluoroalkyl side chains shows complete switching between homeotropic and homogeneous alignments, each of which remains persistent up to its melting point.

## Graphical abstract

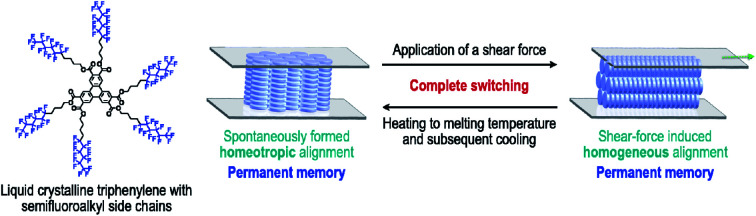

## Introduction

Liquid crystals (LCs) that possess both orientational order and fluidity have high potential as organic functional materials and have been used in many practical applications including displays,^1^ tunable filters,^2^ lenses^3^ and so forth.^4^ One of the greatest advantages of LCs over other materials is that the molecular orientation can be controlled, resulting in anisotropic alignment and properties on the macroscopic scale.^5^ Hence, along with the design of liquid-crystalline molecules with particular properties, considerable effort has been devoted to the development of efficient methods for achieving anisotropic alignment over a large area. Nematic LCs, which have almost no structural correlation among the constituent molecules and thus exhibit low viscosity and high fluidity, can easily be aligned unidirectionally by applying external stimuli such as an electric or magnetic field, mechanical shearing, or surface patterning and modification.^6^ Nonetheless, the degree of orientation that can be achieved with nematic LCs is not particularly high.^7^ Higher-order smectic LCs and columnar LCs, in which the constituent molecules are assembled with two-dimensional (2D) structural correlations, inherently feature excellent orientational order microscopically, whereas their high viscosity due to the low motility of constituent molecules makes macroscopic orientational control extremely difficult.^8,9^ This problem is particularly critical for discotic columnar LCs with a mesogen composed of a large planar π-conjugated molecule.^5*a*,*c*,*d*,6*f*,9^

Discotic columnar LCs can exhibit homeotropic and homogeneous alignments, which are characterized by a structure with one-dimensional (1D) columns oriented perpendicular and parallel, respectively, to the substrate surface,^5*a*,*c*,*d*,6*f*,9^ but in general, discotic LCs exist macroscopically as a mixture of separate domains with these alignments. In the formation of the discotic columnar phase relying on spontaneous molecular assembly on a substrate, homeotropic alignment generally tends to occur preferentially.^5*a*,*c*,*d*,6*f*^ In contrast, unidirectional homogeneous alignment of discotic columnar LCs over the entire material is difficult to achieve and requires special techniques and dedicated instruments as detailed in a comprehensive review article. In this context, Li *et al.* reported an interesting observation regarding switching behavior between homeotropic and homogeneous alignments of a discotic columnar LC by the simple application of a mechanical shear force.^10^ The liquid-crystalline molecule consists of a porphyrin core and four dendritic side chains containing partially perfluoroalkylated (*i.e.*, semifluoroalkyl) groups and exhibits homeotropic alignment upon spontaneous assembly. When a mechanical shear force is applied at a high temperature of the mesophase, the alignment of the LC changes from homeotropic to homogeneous. The resulting homogeneous alignment was reported to be maintained by rapid cooling of the LCs to room temperature, while its retention time and temperature dependence have not been clarified. A fundamental question arises; how does the LC, in which the 1D columns are assembled with a 2D structural correlation, undergo such a macroscopic alignment change while maintaining the microscopic structural order within the column? The dynamics of the columnar assembly during changes in alignment is an interesting issue to explore.

**Fig. 1:**
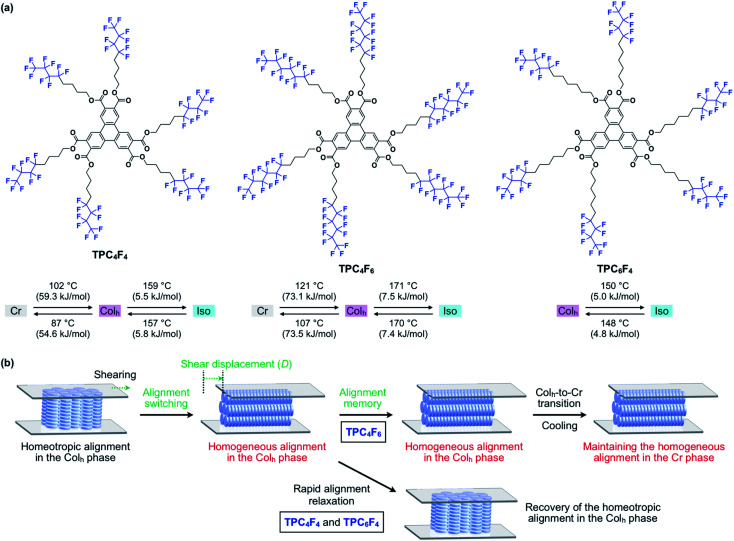
(a) Molecular structures and phase-transition profiles of semifluoroalkylated triphenylene hexacarboxylic ester derivatives TPC_*n*_F_*m*_. (b) Schematic illustrations of the change in orientation in the columnar assemblies of TPC_*n*_F_*m*_ under mechanical shearing. Cr and Iso represent the crystalline phase and isotropic phase, respectively.

Herein, we present the shear force-induced alignment switching of discotic LCs as well as the dynamics of the columnar assembly associated with the change in the alignment, as revealed by the investigation of the phase behaviors of newly designed liquid-crystalline triphenylene-2,3,6,7,10,11-hexacarboxylic esters carrying semifluoroalkyl side chains [–(CH_2_)_*n*_(CF_2_)_*m*–1_CF_3_] with different alkyl/perfluoroalkyl compositions (TPC_*n*_F_*m*_, Fig. 1a). Similar to previously reported alkylated triphenylene hexacarboxylic esters,^11^ the semifluoroalkylated derivatives spontaneously form a large-area homeotropic alignment beyond a centimeter scale. Interestingly, when a mechanical shear force is applied to a triphenylene derivative with semifluoroalkyl (CH_2_)_4_(CF_2_)_5_CF_3_ chains at its mesophase temperature, the entire material undergoes a change in alignment from homeotropic to homogeneous (Fig. 1b). *In situ* X-ray diffraction (XRD) revealed that the columnar assembly turns to be oriented in accordance with the direction of the applied shear force, and the changes in orientation are accelerated when the shear displacement exceeds a certain level. The degree of orientation for the homogeneously aligned LC, determined from transmission X-ray diffraction analysis, was as high as 0.91. This large change in alignment was also supported by polarized optical microscopy (POM) and rheological measurements. The homogeneous alignment of the LC is maintained unless the material is heated to its melting temperature. The memory of alignment is erased by melting, and the large-area homeotropic alignment appears again upon cooling from its isotropic melt.

## Results and discussion

### Molecular design

In 2012, we reported that triphenylene hexacarboxylic ester provides a new mesogen for discotic columnar LCs exhibiting homeotropic alignment over the entire LC film on various substrates.^11^ Although triphenylene is a representative mesogen for creating discotic columnar LCs, all the derivatives that had been reported up to that time had side chains connected *via* ether or thioether linkages. The design concept of the hexacarboxylic ester derivatives was (1) to suppress electrostatic repulsion upon π-stacking by reducing the electron density of the aromatic core with electron-withdrawing ester substitution,^12^ resulting in higher-order columnar assembly, and (2) to enhance intracolumnar and/or intercolumnar structural correlation through dipole–dipole interactions among the ester groups. This concept was successfully demonstrated and led to the development of LCs (TPC_*n*_, Fig. S1, ESI†), which exhibit highly ordered 1D columns, exceptionally long-range 2D structural correlation, and, unexpectedly, perfect homeotropic alignment on glass, metals, metal oxides and even polymer substrates.^11^ Meanwhile, due to their high structural order, the triphenylene hexacarboxylic ester LCs with alkyl side chains are highly viscous, making it difficult to induce a change in alignment, as in the case of usual discotic columnar LCs.

We were interested in how the phase behavior of triphenylene hexacarboxylic ester LCs changes when perfluoroalkyl chains with a larger excluded volume and weaker intermolecular forces^13^ than alkyl chains are introduced.

Semifluoroalkyl side chains have often been used in the design of smectic LCs with rod-shaped molecules to enhance microphase separation, thereby leading to improved structural order of the layered assemblies.^14^ Semifluoroalkyl chains have also been used in the design of columnar LCs, although the number of examples is limited.^9*c*,*f*,15^ For example, in 1996, Percec *et al.* reported that columnar assemblies consisting of tapered dendrons featuring a crown ether and semifluoroalkyl as the tip and peripheral groups, respectively, can align homeotropically on a glass substrate.^16^ Shimizu *et al.* showed that triphenylenes with semifluoroalkyl side chains spontaneously form homeotropic alignment in their Col_h_ LC phases on polyimide-, cetyltrimethylammonium bromide (CTAB)- and InSnO_2_ (ITO)-coated glass substrates.^9*c*^ Including other examples, the preference for the homeotropic alignment in the spontaneous formation of columnar LCs with semifluoroalkyl side chains has been explained as a fluorophobic effect, which qualitatively means an effect to minimize the contact area between fluorinated groups and the substrate surfaces that have in general polar functionalities.

Based on the above-mentioned characteristics of triphenylene hexacarboxylic ester and semifluoroalkyl chains, we designed three semifluoroalkylated triphenylene hexacarboxylic ester derivatives (TPC_4_F_6_, TPC_4_F_4_ and TPC_6_F_4_, Fig. 1a), featuring a different balance in the carbon-chain length of perfluoroalkyl and alkyl groups.

### Synthesis and characterization

**Fig. 2:**
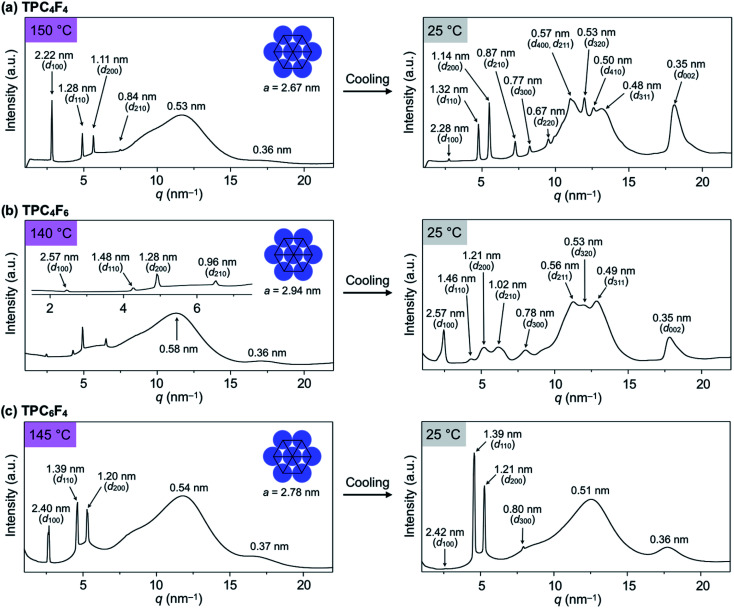
Through-view one-dimensional (1D) XRD patterns of bulk samples of (a) TPC_4_F_4_, (b) TPC_4_F_6_ and (c) TPC_6_F_4_ at their mesophase temperatures (left) and 25 °C (right) in a glass capillary (1.5 mm in diameter) upon cooling from the isotropic liquid phase. Indices of the reflections are shown in parentheses. a.u. = arbitrary units.

TPC_*n*_F_*m*_ (Fig. 1a) were synthesized by the transesterification of triphenylene hexacarboxylic acid methyl ester with the corresponding semifluoroalkyl alcohols using Otera's catalyst and unambiguously characterized by ^1^H/^13^C NMR and FT-IR spectroscopy and APCI-TOF mass spectrometry (ESI†). The phase-transition behaviors of TPC_*n*_F_*m*_ were investigated using differential scanning calorimetry. As shown in Fig. S2 (ESI†), TPC_*n*_F_*m*_ all exhibited a mesophase over a wide temperature range between the crystalline and isotropic phases. The temperatures and enthalpy changes for the phase transitions are summarized in Fig. 1a. The powder X-ray diffraction patterns of TPC_*n*_F_*m*_ recorded at a mesophase temperature (Fig. 2a–c, left) are typical of Col_h_ LC phases. For Instance, TPC_4_F_6_ in its mesophase displayed XRD peaks with *d*-spacings of 2.57, 1.48, 1.28, and 0.96 nm due to diffractions from the (100), (110), (200), and (210) planes of a hexagonal *P*6*mm* structure with a lattice parameter (*a*) of 2.94 nm (Fig. 2b, left). A broad XRD peak arises from columnarly assembled triphenylene units, and its *d*-spacing of 0.36 nm due to diffraction from the (002) plane corresponds to the core-to-core separation. Similarly, the well-defined XRD patterns of the mesophases of TPC_4_F_4_ and TPC_6_F_4_ indicate the formation of 2D hexagonal and 1D columnar assemblies (Fig. 2a and c, left). The structural parameters of Col_h_ phases of TPC_*n*_F_*m*_ are shown in Tables S1–S3 (ESI†). In TPC_6_F_4_, the XRD peaks originating from the 2D lattice are slightly split, which is probably due to the presence of multiple hexagonal structures with slightly different lattice parameters depending on the packing manner of perfluoroalkyl chains in adjacent columns (Fig. 2c, left). Compared with the diffraction peaks due to π-stacked triphenylene at the mesophases of TPC*_n_*, those observed for TPC*_n_*F*_m_* are significantly broader and weaker. This is because the perfluoroalkyl segments with a larger exclusion volume than simple alkyl chains hamper close π-stacking of the triphenylene core, resulting in a decrease in the structural regularity of the 1D columns. Note that, upon cooling from their Col_h_ phases, TPC_4_F_6_ and TPC_4_F_4_ undergo a phase transition into a crystalline phase, which exhibits multiple XRD peaks in a wide-angle region (*q* > 8 nm^−1^), while the diffraction pattern in a small-angle region (*q* < 8 nm^−1^) is almost identical to those observed for the corresponding Col_h_ phases (Fig. 2a and b, right). Moreover, the diffraction peaks due to π-stacked triphenylene in TPC_4_F_6_ and TPC_4_F_4_ became prominent associated with the Col_h_-to-crystal transition. In contrast, TPC_6_F_4_ exhibits no phase transition from the Col_h_ phase to a crystalline phase even upon cooling to a low temperature (*e.g.*, −50 °C, Fig. 2c, right).

**Fig. 3:**
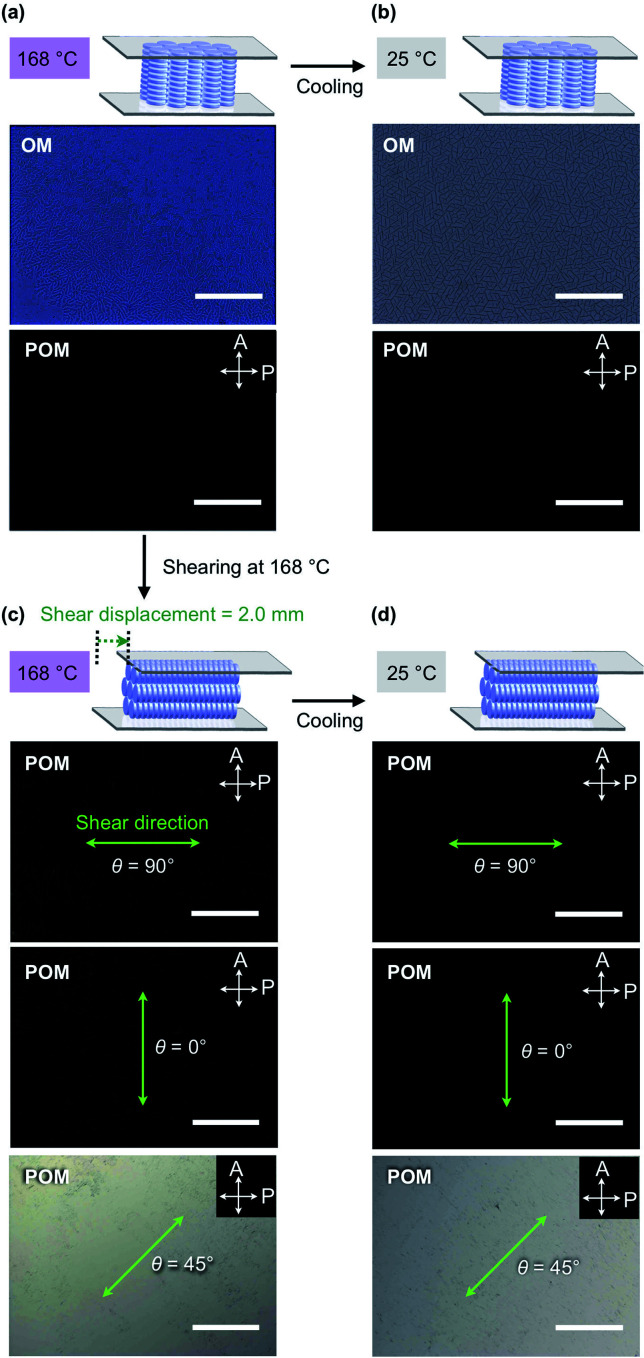
POM and OM images at (a) 168 °C and (b) 25 °C of an 8 μm-thick film of TPC_4_F_6_ sandwiched between sapphire substrates. The film was once heated to the melting point of TPC_4_F_6_ and subsequently cooled to the corresponding temperatures. (c) POM and OM images at 168 °C of an 8 μm-thick film of TPC_4_F_6_ after mechanical shearing at 168 °C (shear displacement = 2.0 mm) and (d) those observed after cooling to 25 °C. White arrows represent the transmission axes of the polarizer (P) and analyzer (A), and green arrows and *θ* represent the shear direction and angles relative to the transmission axis of the analyzer, respectively. Cooling rate: 0.5 °C min^−1^ for the temperature range of the LC mesophase and >30 °C min^−1^ for the temperature range of the crystal phase. Scale bars = 200 μm.

When a film sample of TPC_4_F_6_ (8 μm thick), prepared by sandwiching between sapphire substrates, was once heated to its isotropic melt and then cooled slowly to 168 °C (0.5 °C min^−1^), it displayed an entirely dark image in POM under cross-polarized conditions (Fig. 3a, bottom). The corresponding optical microscopy (OM) image showed a dendritic texture (Fig. 3a, top). These POM and OM features, which are typical of homeotropically aligned hexagonal columnar LCs, were unchanged after the sample was allowed to cool to 25 °C, indicating that the homeotropic alignment is maintained in the crystalline phase (Fig. 3b).^17^ Likewise, film samples of TPC_4_F_4_ and TPC_6_F_4_ were found to exhibit a homeotropic alignment over the entire film (Fig. S3 and S4, ESI†). During the microscopic observations, we happened to notice an intriguing phenomenon: when a shear force is applied from above a sandwiched film of TPC_4_F_6_ in the Col_h_ phase, the POM image changes from a dark field to a bright one. Although a similar phenomenon is often seen in nematic and low-viscosity smectic LCs composed of rod-shaped molecules, it is quite unusual for higher-order LCs including discotic Col_h_ LCs.

### Shear force-responsive switching and memory of columnar alignments

**Fig. 4:**
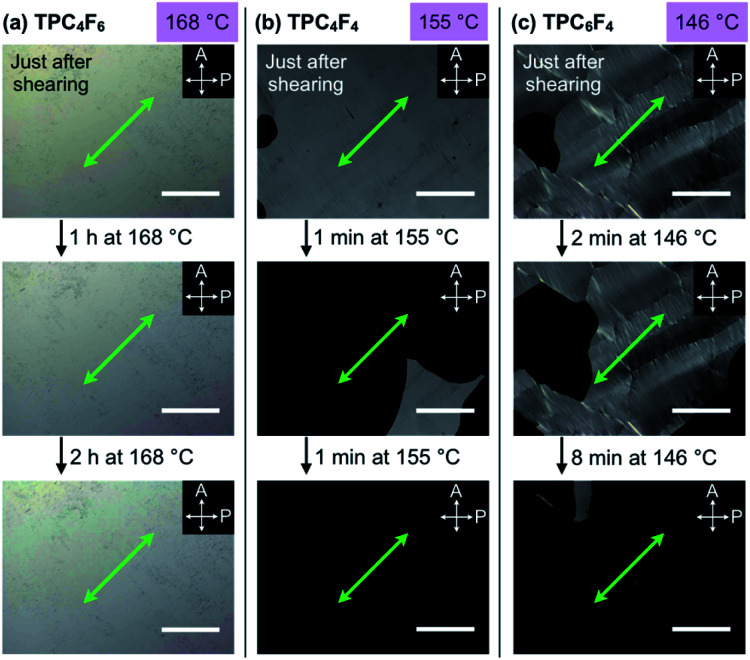
POM images of an 8 μm-thick film of (a) TPC_4_F_6_ at 168 °C just after mechanical shearing (top) and then left standing for 1 h (middle) and 3 h (bottom), (b) TPC_4_F_4_ at 155 °C just after mechanical shearing (top) and then left standing for 1 min (middle) and 2 min (bottom), and (c) TPC_6_F_4_ at 146 °C just after mechanical shearing (top) and then left standing for 2 min (middle) and 10 min (bottom). Each sample was sandwiched between sapphire substrates, once heated to its melting point, cooled to (a) 168, (b) 155, and (c) 146 °C (cooling rate: 0.5 °C min^−1^), and then subjected to a shear force (shear displacement = 2.0 mm) at the measurement temperatures. White arrows represent the transmission axes of the polarizer (P) and analyzer (A), and green arrows represent the shear direction. Scale bars = 200 μm.

To investigate the unexpected shear force-responsive behavior of TPC_4_F_6_ in more detail, we observed the change in the POM image of an 8 μm-thick film of TPC_4_F_6_ sandwiched with round sapphire substrates (*ϕ* = 2.0 cm) while applying a uniaxial shearing force. We kept the temperature of the film at 168 °C (temperature of the Col_h_ phase) and displaced the upper sapphire substrate by 2 mm while applying a shear force. When the polarizer was arranged parallel or perpendicular to the shear direction (*i.e.*, *θ* = 0 or 90°, respectively), the resulting POM image in each case was completely dark (Fig. 3c).^11,17^ When the sample stage was rotated by 45°, the POM image turned to a bright field (Fig. 3c, *θ* = 45°). These observations indicate that the homeotropically aligned columns of TPC_4_F_6_ before shearing change uniformly into a homogeneous alignment after shearing. The same change in alignment induced by a shear force was observed in a wide temperature range of the Col_h_ phase of TPC_4_F_6_. Note that no change in the POM feature was observed, even when a film sample of TPC_4_F_6_, which exhibited a bright POM image due to homogeneous alignment, was allowed to stand at 168 °C for 3 h (Fig. 4a) or was rapidly cooled to room temperature where the crystalline phase of TPC_4_F_6_ appears (Fig. 3d). The homogeneous alignment, once achieved in the Col_h_ phase, was robust enough to be maintained for at least 6 months at 25 °C without relaxation. We would like to emphasize that the phase behavior of TPC_4_F_6_ involving shear force-induced alignment switching and long-lasting memory is exceptional and has not been observed for common rod-like LCs and discotic LCs. Moreover, film samples of TPC_4_F_6_ sandwiched with other substrate materials including glass/glass, quartz/quartz, mica/mica, glass/Teflon, and polyimide/sapphire showed a similar shear force-responsive behavior (Fig. S5, ESI†). We examined the thickness-dependence of the alignment switching/memory behavior of TPC_4_F_6_ and confirmed that the same behavior can be achieved at least for a film with thicknesses of up to 20 μm (Fig. S6 and S7, ESI†).

As in the case of TPC_4_F_6_, a film of TPC_4_F_4_ showed a change in alignment from homeotropic to homogeneous upon application of a shear force in the temperature range of its Col_h_ phase (*e.g.*, 155 °C). However, the resulting homogeneous alignment quickly relaxed to regenerate the original homeotropic alignment, where the retention time was approximately 1 minute at 155 °C (Fig. 4b). Although a film of TPC_6_F_4_ also showed a change in alignment when a shear force was applied at its Col_h_ phase temperature (*e.g.*, 146 °C), the change occurred only partially, and the resulting homogeneously aligned domains were quickly returned to the original homeotropic alignment (Fig. 4c). Considering all the observations for TPC_n_F_*m*_, the balance between the lengths of alkyl and perfluoroalkyl chains comprising the side chains is critical for the shear-force induced alignment switching/memory behavior as well as the thermal stability of the two alignments.

**Fig. 5:**
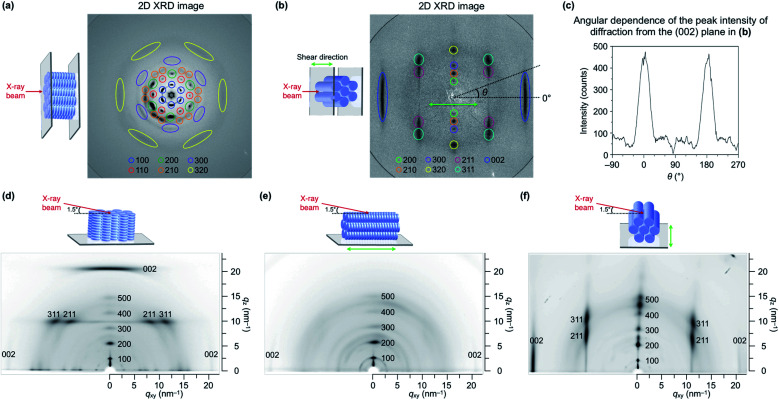
Through-view two-dimensional (2D) XRD images at 25 °C of 8 μm-thick films of TPC_4_F_6_ sandwiched between sapphire substrates, (a) prepared by cooling from its melting point to 25 °C and (b) prepared by cooling from its melting point to 168 °C, sheared at the same temperature (shear displacement = 2.0 mm and shear rate = 1.5 μm s^−1^), and then cooled to 25 °C. In (b), the green arrow represents the shear direction. Cooling rate: 0.5 °C min^−1^ for the temperature range of the LC mesophase and >30 °C min^−1^ for the temperature range of the crystal phase. (c) Angular (*θ*) dependence of the peak intensity of diffraction from the (002) plane, obtained by converting the 2D XRD image in (b). (d–f) 2D GI-XRD images at 25 °C of 8 μm-thick films of TPC_4_F_6_ on a sapphire substrate measured after removing the upper sapphire substrate from the corresponding sandwiched films. The sample for (d) was prepared according to the protocol described in (a). The samples for (e and f) were prepared according to the protocol described in (b). The directions of an incident X-ray beam for (e) and (f) were parallel and perpendicular to the shear direction, respectively, where the incident angle of the X-ray beam was 0.15°.

We performed transmission (through-view) and grazing-incidence (GI) XRD measurements to gain further insight into the molecular orientation of TPC_4_F_6_ in its film. When a film of TPC_4_F_6_ sandwiched between sapphire substrates without a shear-force treatment was exposed at 25 °C to an incident X-ray beam in the direction perpendicular to the film surface, diffraction spots with 6-fold symmetry appeared (Fig. 5a). This is consistent with the microscopic observations that crystalline TPC_4_F_6_ virtually inherits the structure as well as the homeotropic alignment of the Col_h_ mesophase.^11^ In contrast, when a through-view XRD image was similarly recorded for a film of TPC_4_F_6_ after a shear-force treatment at a temperature of its Col_h_ phase (shear displacement = 2.0 mm), groups of linearly aligned diffraction spots were observed, in addition to linear-shape diffractions in a wide-angle region, due to π-stacked triphenylene (Fig. 5b). The linearly aligned diffraction spots can be assigned to the (200), (210), (300), (320), (211), (311), and (002) planes of a hexagonal *P*6*mm* structure. The results obtained by through-view XRD measurements clearly show that the application of a shear force changes the alignment of columns uniformly from homeotropic to homogeneous. Moreover, we confirmed that through-view XRD images of a homogeneously aligned TPC_4_F_6_ film measured at various different positions are identical to one another, without causing displacement of the positions where the diffraction spots appear. This means that the film of TPC_4_F_6_ is highly uniform so that domain boundaries cannot be detected.

The change in the orientation of TPC_4_F_6_ was also confirmed by GI-XRD measurements. In the GI-XRD image of a film of TPC_4_F_6_ on a sapphire substrate without a shear-force treatment, the diffraction spot derived from the (002) plane clearly appeared in the out-of-plane direction (Fig. 5d). When a shear-force was applied to the film, the diffraction spot appeared in the in-plane direction (Fig. 5e and f). Obviously, the shear-force treatment changes the direction of the longer axis of the π-stacked triphenylene column from perpendicular to parallel relative to the substrate surface.

Similar to TPC_4_F_6_, sandwiched films of TPC_4_F_4_ and TPC_6_F_4_ showed a shear force-responsive homeotropic-to-homogeneous change in alignment at the mesophase temperature, as confirmed by the through-view and GI-XRD measurements (Fig. S8–S10, ESI†). However, as observed by POM (Fig. 4b and c), the resultant homogeneous alignment of TPC_4_F_4_ and TPC_6_F_4_ rapidly reverted to the homeotropic alignment at their mesophase temperatures. The homogeneous orientation for TPC_4_F_4_ can be maintained by cooling the sample to 25 °C (*i.e.*, crystalline phase) immediately after shear-force treatment. The contrasting shear force-responsive orientation behaviors between TPC_4_F_6_ and TPC_4_F_4_/TPC_6_F_4_ are interesting and highlight the importance of side-chain design in terms of the balance of alkyl and fluoroalkyl segments.

Notably, the order parameter (*S*) of the homogeneously aligned film of TPC_4_F_6_, calculated from the diffraction due to π-stacked triphenylene, was as high as 0.91 (Table 1 and Fig. 5c). This value is comparable to those achieved with highly oriented rod-like LCs^8^ and much higher than those reported for existing discotic LCs.^18^ Meanwhile, the value of *S* based on the optical anisotropy evaluated by polarized UV spectroscopy was 0.30 (Table 1 and Fig. S12, ESI†), which is much smaller than that based on the structural anisotropy evaluated by XRD. We consider that unlike rod-shaped molecules featuring a uniaxial transition dipole moment and a large dichroic nature, in discotic triphenylene with 3-fold symmetry, the influence of molecular orientation on the angle dependence of polarized light absorption would be small. It seems inherently difficult to achieve high optical anisotropy with discotic LCs composed of a mesogenic core with a small dichroic nature.

**Table 1:** Order parameters for homogeneously aligned films of TPC_*n*_F_*m*_ estimated from through-view 2D XRD analysis (*S*_π_) and polarized electronic absorption spectroscopy (*S*)^a^

	TPC_4_F_6_	TPC_4_F_4_	TPC_6_F_4_
*S*_π_	0.91	0.92^b^	n.d.^b^^,^^c^
*S*	0.30	0.36^d^	0.24^d^

a Measured at 25 °C. Samples were prepared by mechanical shearing at each mesophase temperature and then cooling to 25 °C.

b For the experimental data, see Fig. S8–S10 (ESI).

c Not determined due to the absence of a diffraction peak arising from the core-to-core separation.

d For the experimental data, see Fig. S11 and S13 (ESI).

**Fig. 6:**
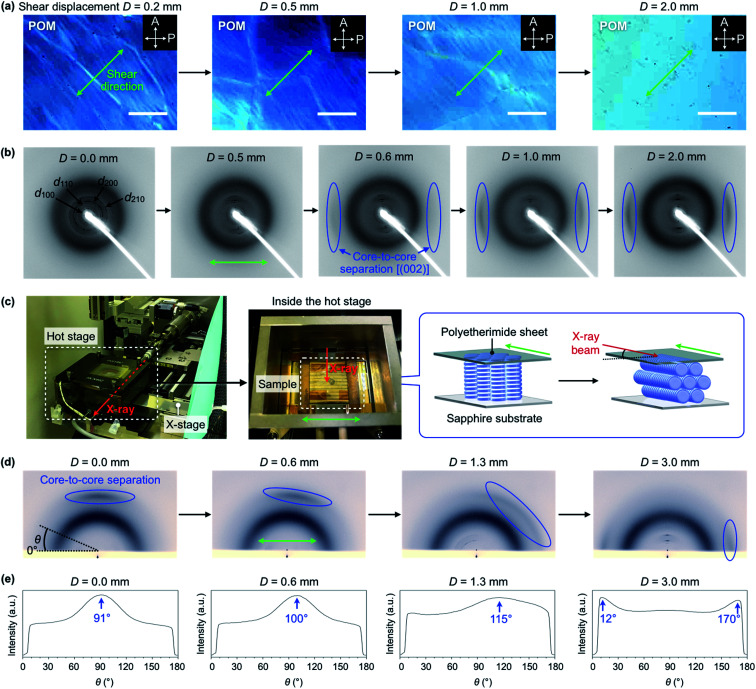
*In situ* POM and XRD measurements under the application of mechanical shearing (shear rate = 1.5 μm s^−1^). The samples were prepared by sandwiching TPC_4_F_6_ between the corresponding solid substrates, once heated to its melting point, and then cooled to 168 °C (cooling rate: 0.5 °C min^−1^). (a) POM images (at 168 °C) of an 8 μm-thick film of TPC_4_F_6_ sandwiched between sapphire substrates. The sample was tilted at 45° relative to the transmission axis of the polarizer. Scale bars = 200 μm. (b) Through-view 2D XRD images (at 168 °C) of a 10 μm-thick film of TPC_4_F_6_ sandwiched between sapphire substrates. The blue circles indicate diffraction from the (002) plane (*i.e.*, core-to-core separation). The experimental setups for the *in situ* POM and through-view XRD measurements are shown in Fig. S14a and S14b,† respectively. (c) Photographs and schematic representations of a dedicated experimental setup for *in situ* reflective XRD measurements (incident angle = 1.5°). (d) Reflective 2D XRD images (at 168 °C) of a 10 μm-thick film of TPC_4_F_6_ sandwiched between a polyetherimide sheet and a sapphire substrate. (e) Angular (*θ*) dependence of the peak intensity of diffraction from the (002) plane, obtained by converting the 2D XRD images in (d). The green arrows represent the shear direction. Full data sets for the reflective XRD (Fig. S16, ESI), as well as those for the through-view XRD (Fig. S15, ESI), are provided in the ESI.†

To understand the mechanism of the exceptional alignment behavior of TPC_4_F_6_, we performed a quantitative investigation of the effect of shear displacement on the column orientation under the application of a shear force at a constant rate. Fig. 6a shows changes in a POM image of a homeotropically aligned TPC_4_F_6_ film at 168 °C upon continuous shearing at a rate of 1.5 μm s^−1^ (see also Fig. S14a for the experimental setup, ESI†). As the displacement was increased, the POM image gradually changed from dark to bright. At a shear displacement (*D*) of 2.0 mm, the entire film gave a completely white image (Fig. 6a), and the POM image no longer changed upon further shearing. Interestingly, during the application of shear force up to a displacement of 2.0 mm, a white string-like texture appeared in POM. With an increase in the number of string-like textures, the POM image became brighter and brighter, resulting in the final white image. It is reasonable to consider that the string-like texture represents homogeneously aligned domains, while the dark-field regions are due to remaining homeotropic alignment domains.

Using a dedicated experimental setup that allows *in situ* XRD measurements while applying a shear force (Fig. S14b, ESI†), we investigated the relationship between shear displacement and the orientation of columns inside a film of TPC_4_F_6_. Fig. 6b and S15 (ESI†) show through-view XRD images obtained upon exposure of a homeotropically aligned film sandwiched between sapphire substrates to an X-ray beam at 168 °C while shearing at a rate of 1.5 μm s^−1^. When the displacement (*D*) reached approximately 0.6 mm, diffraction arcs arising from π-stacked triphenylene began to appear (Fig. 6b). On the other hand, diffraction spots from the (100), (110), (200) and (210) planes of the hexagonal lattice were converged into the direction perpendicular to the shear direction. Thus, the through-view XRD images allowed direct observation of how the triphenylene column, which had been oriented perpendicular to the substrate surface, changed to a homogeneous orientation. In reflective XRD measurements (Fig. 6c, d and S16, ESI,† incident angle = 1.5°), the diffraction arcs arising from π-stacked triphenylene, which had been observed in the out-of-plane direction when *D* = 0.0 mm, gradually moved to the in-plane direction (Fig. 6d, e and S16, ESI†). Interestingly, in the shearing process until the displacement reaches up to approximately 1.7 mm, these diffraction arcs were once extended and then converged in the in-plane direction (Fig. 6d, e and S16, ESI†). Considering the fact that the total intensity and half width of the peak are hardly changed during the extension/convergence process of the diffraction arcs, the switching of columnar orientation likely proceeds while mostly preserving the microscopic molecular arrangement of the π-stacked triphenylene inside the column.

The change in the XRD images agrees well with the POM observations (Fig. 6a), where the string-like texture appears and increases, and eventually disappears, during the change in alignment from homeotropic to homogeneous. Based on the above results, we consider that the assemblies of TPC_4_F_6_ in the film are divided into elongated string-like domains upon application of a shear force, and each domain has motility to some extent, and thereby undergoes a gradual change from perpendicular to homogeneous orientations with respect to the substrate surface. Once a homogeneous alignment is achieved, string-like domains are fused into a large and uniform domain to lose motility. As a consequence, the memory of the homogeneous alignment is retained without recovering the thermodynamically favorable homeotropic alignment, unless the material is heated to its isotropic melt.

### Rheological properties

**Fig. 7:**
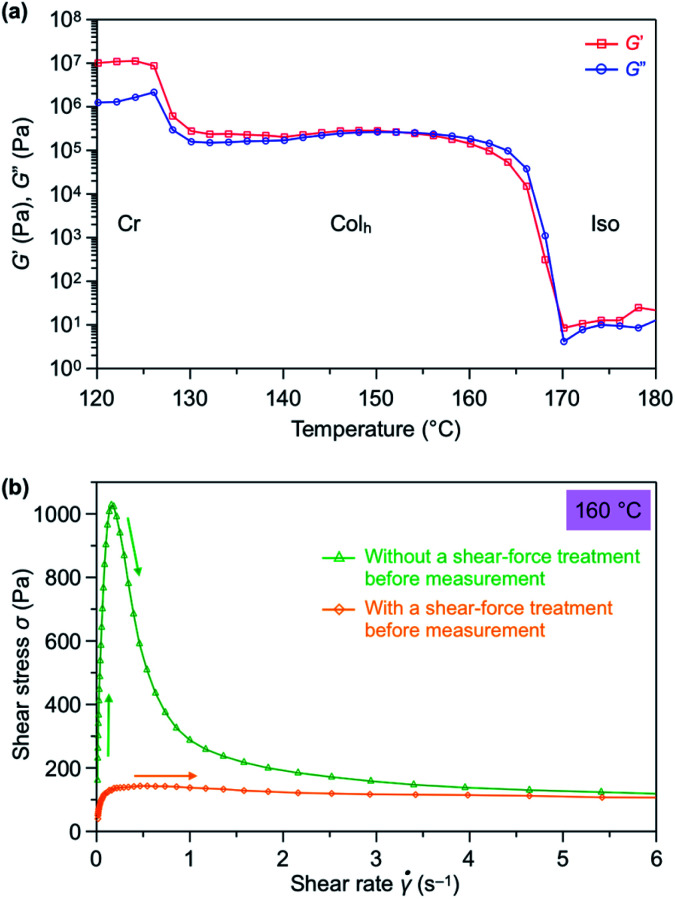
Rheological properties of TPC_4_F_6_. (a) Temperature-dependence (in a heating process) of the storage modulus (*G*′; red) and loss modulus (*G*′′; blue) under an applied strain (*γ*) of 0.1% and an angular frequency (*ω*) of 1.0 Hz. (b) Shear rate-dependence of shear stress at 160 °C without (green) and with (orange) shear-force treatment before measurement.

We hypothesized that the exceptional ability of TPC_4_F_6_ to completely change from a homeotropic alignment, which occurs by its self-assembly, to a homogeneous alignment by the application of a shear force may originate from the rheological properties of TPC_4_F_6_. Using a rotational rheometer, we measured the dynamic viscoelasticity of TPC_4_F_6_ in a heating process at a shear rate (*γ*) of 0.1%, frequency (ω) of 1 Hz, and cell gap (*d*) of 100 μm. Interestingly, the storage (*G*′) and loss (*G*′′) elastic moduli observed for the Col_h_ phase of TPC_4_F_6_ were comparable to one another in the heating process (Fig. 7a).^19^ Such viscoelastic behavior is rarely observed for ordinary soft materials, except for a previously reported triphenylene hexacarboxylic ester derivative carrying chiral alkyl chains.^20^

The peculiar rheological properties of TPC_4_F_6_ can be qualitatively interpreted as being a result of the fact that TPC_4_F_6_ has both solid- and liquid-like properties to the same extent, which may provide this material with a softness leading to a shear force-responsive ability as well as a hardness leading to long-standing alignment memory. Furthermore, when the shear stress (*σ*) for a film with homeotropically aligned columns was measured at 160 °C while varying the shear rate in the range of 0.01–10 s^−1^, *σ* shows the maximum value at a shear rate of 0.16 s^−1^ (Fig. 7b), in which the shear displacement at half the radius from the center of the parallel-plate-type jig was 1.17 mm and then sharply decreased.^21^ This indicates that while the homeotropic alignment of TPC_4_F_6_ can be preserved at a certain level of strain due to its elasticity, further shearing induces a change in orientation, resulting in the homogeneous alignment. The phenomenon that the orientation change occurs discontinuously when shear displacement exceeds a certain level is consistent with the results of the XRD measurements. Once the material changed into a homogeneous alignment by mechanical shearing, the values of shear stress dropped to a low level. When the shear rate exceeded 2 s^−1^, the values of shear stress became comparable to those observed for a separately prepared film of TPC_4_F_6_ with a homogeneous alignment, where the values of shear stress are almost unchanged over a wide range of shear rates.

The dynamic viscoelasticity profile of TPC_4_F_4_ also showed comparable *G*′ and *G*′′ values (Fig. S17a, ESI†). However, the shear rate-dependence of shear stress at its mesophase (150 °C) was largely different from that observed for TPC_4_F_6_, where the values of shear stress of TPC_4_F_4_ did not significantly decrease with increasing shear rate (Fig. S17b, ESI†). Moreover, the values of shear stress observed for a film sample of TPC_4_F_4_, separately prepared by a shear-force treatment, were greater than those observed for TPC_4_F_6_ over a wide range of shear rates (Fig. S17b, ESI†). The difference in rheological properties between TPC_4_F_6_ and TPC_4_F_4_ may reflect their orientational behaviors: the former can retain the memory of the shear force-induced homogeneous alignment unless the material is heated to its melting temperature, while this does not hold true for the latter.

Based on all of the above experimental results, we discuss the shear force-induced alignment switching behavior of TPC_4_F_6_. In the Col_h_ phase of TPC_4_F_6_, the structural order of the π-stacked triphenylene is low, due to the presence of the perfluoroalkyl segment with a large exclusion volume in the side chains. Given that short perfluoroalkyl chains have weak assembly forces,^13*e*^ the interaction between adjacent columns for which the outer surface is covered with perfluoroalkyl chains should be small. Hence, both 1D columnar and 2D hexagonal assemblies most likely have structural fluctuations to the extent that the phase separation between the alkyl segments, in which dispersion force dominantly occurs, and the perfluoroalkyl segments, in which dipole–dipole interactions due to the polarization of C–F bond dominantly occur, is not impaired. The structural fluctuations would be closely related to the rheological properties of TPC_4_F_6_ in the Col_h_ mesophase where *G*′ and *G*′′ are comparable to one another, despite it being a type of discotic columnar LC. The exceptional rheological properties with a fine balance between storage and loss elastic moduli are considered to be responsible not only for the occurrence of the string-like homogeneously aligned fluid domains, but also for their fusion into a larger and uniform domain that is insusceptible to structural relaxation over a wide temperature range.

## Conclusions

Controlling the alignment of discotic columnar LCs remains a difficult task. If this can be achieved with molecular design coupled with a readily accessible method, it could be useful to take best advantage of the intrinsic properties of the materials for many applications. As a clue for how to tackle this issue, we refer to a paper by Li *et al.*, who reported a phenomenon in which a porphyrin derivative with dendritic side chains containing perfluoroalkyl groups shows a change in alignment from homeotropic to homogeneous upon shearing, whereas no further details have been reported.

Through the detailed investigation of the structure, phase and alignment behaviors and rheological properties of newly synthesized discotic liquid-crystalline molecules with a triphenylene hexacarboxylic acid ester as the mesogen and semifluoroalkyl groups as the side chains (TPC_*n*_F_*m*_), we demonstrated that materials design using perfluoroalkylated side chains holds promise for controlling the alignment of discotic columnar LCs. The key finding is that one of the derivatives (TPC_4_F_6_) exhibits a homeotropically aligned hexagonal columnar (Col_h_) mesophase upon self-assembly under thermodynamic control on various substrates, while upon the application of a shear force in the mesophase, the homeotropic alignment switches completely into a homogeneous alignment, where the column is oriented unidirectionally along the direction of shear. Notably, the homogeneous alignment is maintained over a wide temperature range. We consider that the excellent ability of triphenylene hexacarboxylic acid ester to facilitate exceptionally long-range uniform molecular ordering certainly plays a role in the unique behavior of TPC_4_F_6_, but more importantly, this material has comparable storage elastic and loss elastic moduli, most likely originating from the perfluoroalkyl groups covering the outer surface of the 1D column. We believe that the present finding could provide new design guidelines for the development of discotic LCs that would enable the flexible control of molecular alignment to suit a particular purpose.

## Data availability

All experimental data associated with this work are available in the ESI.†

## Author contributions

T. F. conceive the project; T. K., Y .S. and T. F. designed the experiments; M. K., A. K., Y. S. and T. K. carried out the synthesis and characterization of the materials; M. K., R. H., R. K., S. A. and T. K. performed the X-ray diffraction experiments and analyzed the data; M. K., Y. S. and T. K. performed the rheological measurements and analyzed the data; Y. S., T. K. and T. F. co-wrote the manuscript.

## Conflicts of interest

There are no conflicts to declare.

## Supplementary Material

SC-013-D2SC03677K-s001
