# Supplementary material for: Design of discotic liquid crystal enabling complete switching along with memory of homeotropic and homogeneous alignment over a large area
Source: Chem Sci. 2022 Jul 19;13(34):9891–901. doi: 10.1039/d2sc03677k (PMC9430577; doi:10.1039/d2sc03677k)
Supplement: SC-013-D2SC03677K-s001 [file SC-013-D2SC03677K-s001.pdf]

Electronic Supplementary Material (ESI) for Chemical Science.
This journal is © The Royal Society of Chemistry 2022

Supplementary Information

Design of discotic liquid crystal enabling complete switching between and memory of two alignment states over a large area

Yoshiaki Shoji, Miki Kobayashi, Atsuko Kosaka, Rie Haruki, Reiji Kumai, Shin-ichi Adachi, Takashi Kajitani* and Takanori Fukushima*

E-mail: fukushima@res.titech.ac.jp (T.F.), kajitani.t.ab@m.titech.ac.jp (T.K.)

Table of Contents

1. Materials S2
2. Methods S2
3. Synthesis S3
4. Powder X-ray diffraction (XRD) analysis S4
5. Rheology measurements S4
6. Supplementary references S5
7. Supplementary tables (Tables S1–S3) S6
8. Supplementary figures (Figs. S1–S17) S7

1. Materials

Unless otherwise stated, all commercial reagents were used as received. 1-Chloro-3-hydroxy-1,1,3,3-tetrabutyldistannoxane[S1] and 2,3,6,7,10,11-hexa(ethoxycarbonyl)triphenylene (**TPC_2**)[S2] were prepared according to previously reported procedures. Solid substrates were purchased from Matsunami Glass Ind., Ltd. (glass), Sigma Koki Co., Ltd. (sapphire, ϕ = 2.0 cm), Electronics and Materials Co., Ltd. (silicon wafer, 4PO type), Nilaco Corp. (Kapton film), Teraoka Seisakusho Co., Ltd. (Kapton tape®), and Mitsubishi Chemical Corp. (SUPERIO™ UT). Column chromatography was carried out using Wakogel silica C-300 (particle size: 45–75 μm).

2. Methods

Preparative size-exclusion chromatography (SEC) was carried out on a Japan Analytical Industry LC-9201 recycling preparative HPLC system, equipped with JAIGEL-1H and JAIGEL-2H columns and a multi-wavelength detector (MD-2010$_{Plus}$), using $CHCl_3$ as an eluent. NMR spectroscopy measurements were carried out on a Bruker AVANCE III HD-500 spectrometer (500 MHz for ^{1}H, 125 MHz for ^{13}C and 471 MHz for ^{19}F). Chemical shifts (δ) are expressed relative to the resonances of the residual non-deuterated solvent for ^{1}H [$CDCl_3$: $^{1}H(\delta)$ = 7.26 ppm] and ^{13}C [$CDCl_3$: $^{13}C(\delta)$ = 78.0 ppm]. For ^{19}F NMR measurements, external standards were used: trifluoroacetic acid in $CDCl_3$ for ^{19}F [^{19}F (δ) = –76.5 ppm]. Absolute values of the coupling constants are given in Hertz (Hz), regardless of their sign. Multiplicities are abbreviated as singlet (s), triplet (t) and multiplet (m). APCI-TOF mass spectrometry measurements were carried out on a Bruker micrOTOF II mass spectrometer equipped with an atmospheric pressure chemical ionization (APCI) probe. Fourier-transform infrared (FT-IR) spectra were recorded at 25 °C on a JASCO FT/IR-660$_{Plus}$ Fourier-transform infrared spectrometer. Differential scanning calorimetry (DSC) measurements were carried out on a Mettler–Toledo DSC 1 differential scanning calorimeter, where temperature and enthalpy were calibrated with In (430 K, 3.3 J/mol) and Zn (692.7 K, 12 J/mol) standard samples in sealed Al pans. Cooling and heating profiles were recorded and analyzed using the Mettler−Toledo STAR^{e} software system. Polarized optical microscopy (POM) was performed on a Nikon Eclipse LV100POL optical polarizing microscope, equipped with a Mettler–Toledo HS1 controller attached to a HS82 hot stage. Optical retardation measurements were performed on a Nikon Eclipse LV100POL optical polarizing microscope, equipped with a Sénarmont compensator at a wavelength of 546 nm. Surface profile measurements of horizontally aligned films on a quartz substrate were carried on a Dektak XT surface profiler. Polarized electronic absorption spectra were recorded on a JASCO V-670 UV/VIS spectrometer equipped with JASCO RSH-744 rotation sample holder and JASCO GPH-506 polarizer.

3. Synthesis

TPC_4F_4. Under argon, a trifluorotoluene solution (15 mL) of a mixture of **TPC_2** (200 mg, 0.30 mmol), 2-(perfluorohexyl)butanol (852 mg, 2.92 mmol), and 1-chloro-3-hydroxy-1,1,3,3-tetrabutyldistannoxane (71.2 mg, 0.13 mmol) was refluxed for 24 h, where generated water was removed using a Dean-Stark trap. The reaction mixture was allowed to cool to room temperature, and then evaporated to dryness under reduced pressure. The residue was subjected to column chromatography on SiO_2 (CH_2Cl_2/AcOEt, v/v = 100/1), followed by recycling preparative SEC (JAIGEL 1H/2H) with $CHCl_3$ as an eluent. Fractions containing **TPC_4F_4** were collected and evaporated to dryness under reduced pressure. The residue was recrystallized from hexane to give **TPC_4F_4** (569 mg, 0.27 mmol) as a white powder in 88% yield. ^{1}H NMR (500 MHz, $CDCl_3$, 25 °C): δ (ppm) 9.02 (s, 6H), 4.47 (t, J = 6.4 Hz, 12H), 2.23–2.12 (m, 12H), 1.97–1.91 (m, 12H), 1.84–1.78 (m, 12H). ^{13}C NMR (125 MHz, $CDCl_3$, 25 °C): δ (ppm) 167.11, 131.84, 131.09, 125.55, 118.65, 118.55, 118.31, 118.05, 116.36, 65.602, 30.52 (t, J = 22.3 Hz, 36C), 28.21, 17.19. ^{19}F NMR (471 MHz, $CDCl_3$, 25 °C): δ (ppm) –81.24 (t, J = 10.5 Hz, 18F), –114.79 to –114.92 (m, 12F), –124.64 to –124.70 (m, 12F), –126.16 to –126.25 (m, 12F). FT-IR (KBr): ν (cm^{-1}) 2963, 2891, 1731, 1618, 1473, 1358, 1286, 1236, 1202, 1135, 719. APCI-TOF mass: calcd. for $C_{72}H_{54}F_{54}O_{12}$ $[M+H]^+$: m/z = 2137.28; found: 2137.31.

TPC_4F_6. Under argon, a trifluorotoluene solution (15 mL) of a mixture of **TPC_2** (150 mg, 0.23 mmol), 2-(perfluorohexyl)butanol (857 mg, 2.19 mmol), and 1-chloro-3-hydroxy-1,1,3,3-tetrabutyldistannoxane (48.9 mg, 0.09 mmol) was refluxed for 24 h under argon, where generated water was removed using a Dean-Stark trap. The reaction mixture was allowed to cool to room temperature, and then evaporated to dryness under reduced pressure. The residue was recrystallized from $CHCl_3$ to give **TPC_4F_6** (354 mg, 0.13 mmol) as a white powder in 57% yield. ^{1}H NMR (500 MHz, $CDCl_3$, 25 °C): δ (ppm) 9.02 (s, 6H), 4.48 (t, J = 6.4 Hz, 12H), 2.23–2.14 (m, 12H), 1.98–1.92 (m, 12H), 1.86–1.80 (m, 12H). ^{13}C NMR (125 MHz, $CDCl_3$, 25 °C): δ (ppm) 167.04, 132.82, 132.07, 131.22, 130.58, 130.38, 130.18, 127.56, 127.36, 127.16, 125.61, 118.57, 116.55, 116.33, 116.09, 111.65, 110.86, 109.26, 108.98, 65.58, 30.85 (t, J = 22.6 Hz, 36C), 28.37, 17.38. ^{19}F NMR (471 MHz, $CDCl_3$, 25 °C): δ (ppm) –81.57 (t, J = 10.3 Hz, 18F), –114.68 to –114.82 (m, 12F), –122.29 to –122.41 (m, 12F), –123.28 to –123.39 (m, 12F), –124.00 to –124.07 (m, 12F), –126.58 to –126.66 (m, 12F). FT-IR (KBr): ν (cm^{-1}) 2962, 2896, 1731, 1616, 1473, 1366, 1286, 1240, 1201, 1146, 1123, 732. APCI-TOF mass: calcd. for $C_{84}H_{54}F_{78}O_{12}$ $[M + H]^+$: m/z = 2737.24; found: 2737.14.

TPC_6F_4. By procedures similar to that for **TPC_4F_4**, **TPC_6F_4** was obtained in 60% yield as a white powder (208 mg, 0.09 mmol) from **TPC_2** (101 mg, 0.15 mmol), 2-(perfluorobutyl)hexanol (476 mg, 1.49 mmol) and 1-chloro-3-hydroxy-1,1,3,3-tetrabutyldistannoxane (32.3 mg, 0.06 mmol). ^{1}H NMR (500 MHz, $CDCl_3$, 25 °C): δ (ppm) 9.03 (s, 6H), 4.43 (t, J = 6.3 Hz, 12H), 2.13–2.02 (m, 24H), 1.88–1.82 (m, 12H), 1.68–1.63 (m, 12H), 1.51–1.49 (m, 12H). ^{13}C NMR (125 MHz, $CDCl_3$, 25 °C): δ (ppm) 167.04, 132.82, 132.07, 131.22, 130.58, 130.38, 130.18, 127.56, 127.36, 127.16, 125.61, 118.57, 116.55,

116.33, 116.09, 111.65, 110.86, 109.26, 108.98, 65.58, 30.85 (t, J = 22.6 Hz, 36C), 28.37, 17.38. ^{19}F NMR (471 MHz, CDCl3, 25 °C): δ (ppm) –80.96 (t, J = 9.9 Hz, 18F), –114.43 to –114.57 (m, 12F), –124.34 to –124.38 (m, 12F), –125.93 to –125.96 (m, 12F). FT-IR (KBr): ν (cm^{-1}) 2951, 2861, 1729, 1617, 1469, 1357, 1282, 1227, 1203, 1135, 1083, 719. APCI-TOF mass: calcd. for $C_{84}H_{78}F_{54}O_{12}$ [M + H]$^+$: m/z = 2305.47; found: 2305.30.

4. Powder X-ray diffraction (XRD) analysis

Synchrotron-radiation XRD measurements. Variable-temperature (VT) powder XRD patterns of bulk samples of **TPC$_n$F$_m$**, through-view XRD images of their film samples, and grazing incidence X-ray diffraction (GI-XRD) images of their film samples were measured using the BL45XU beamline at SPring-8 (Hyogo, Japan) equipped with a Pilatus3X 2M (Dectris) detector. The scattering vector ($q = 4\pi\sin\theta/\lambda$) and the position of the incident X-ray beam on the detectors were calibrated using several orders of layer reflections from silver behenate (d = 58.380 Å), where 2θ and λ refer to the scattering angle and wavelength of the X-ray beam (1.0 Å), respectively. The sample-to-detector distances for powder XRD, through-view XRD, and GI-XRD measurements were 0.33, 0.33, and 0.28 m, respectively. The obtained diffraction patterns were integrated along the Debye-Scherrer ring to afford 1D intensity data using the FIT2D software[S3]. The cell parameters were refined using the CellCalc ver. 2.10 software [S4].

***In situ* XRD measurement in the presence of a linear motorized stage.** *In situ* X-ray diffraction experiments in the presence of a linear motorized stage (x-stage; XA05A-R101, Kohzu Precision Co.) were carried out using the BL-8B beamline at Photon Factory (Ibaraki, Japan). A film sample of **TPC$_4$F$_6$** sandwiched between a sapphire substrate and a polyetherimide sheet was attached to a Instec HTC-402 hot stage, heated once to its melting point, cooled to 168 °C (cooling rate: 2.0 °C/min), and then subjected to a shear force (shear distance = 2.0 mm) at the same temperature (Figure 6b,d). The sample was exposed to the X-ray beam (λ = 1.0 Å) with an incident angle of 90° (Figure 6b) and 1.5° (Figure 6d). The X-ray diffraction images were collected using an imaging-plate area detector.

5. Rheology measurements

Dynamic viscoelasticity measurements. The dynamic viscoelasticity data of **TPC$_4$F$_6$** and **TPC$_4$F$_4$** were obtained using an Anton Paar MCR102 rotational rheometer with a parallel-plate-type jig with a diameter of 20 mm and a sample gap of 100 μm. The samples were once heated to the melting point and subsequently cooled (cooling rate = 2 °C/min) to the measurement temperatures (120 and 90 °C for **TPC$_4$F$_6$** and **TPC$_4$F$_4$**, respectively). The data were collected by increasing the measurement temperature while rotating the jig under an applied strain (γ) of 0.1% and an angular frequency (ω) of 1.0 Hz.

Viscosity measurements. The shear viscosities of **TPC$_4$F$_6$** and **TPC$_4$F$_4$** were measured using an Anton Paar MCR102 rotational rheometer equipped with a parallel-plate-type jig with a

diameter of 20 mm and a sample gap of 20 μm. In the measurements of the LC materials without a shear-force treatment, samples were prepared by once heating to the melting point and then cooling to 25 °C (cooling rate = 2 °C/min). In the measurements of the LC materials with a shear-force treatment, samples were prepared by once heating to the melting point and the cooling to 160 and 150 °C for **TPC$_4$F$_6$** and **TPC$_4$F$_4$**, respectively (cooling rate = 2 °C/min). The resultant samples, after being applied to a shear force at the same temperature by rotating the jig, were cooled to 25 °C (cooling rate = 2 °C/min). The viscosity data were obtained upon heating the samples at the measurement temperatures (160 and 150 °C for **TPC$_4$F$_6$** and **TPC$_4$F$_4$**, respectively). The data were collected by changing the shear rate from small to large values.

6. Supplementary references

S1. J. Otera, N. Danoh and H. Nozaki, *J. Org. Chem.*, 1991, **56**, 5307–5311.

S2. T. Osawa, T. Kajitani, D. Hashizume, H. Ohsumi, S. Sasaki, M. Takata, Y. Koizumi, A. Saeki, S. Seki, T. Fukushima and T. Aida, *Angew. Chem.*, *Int. Ed.*, 2012, **51**, 7990−7993.

S3. A. Hammersley, FIT2D v.17.006 (European Synchrotron Radiation Facility, 2015); http://www.esrf.eu/computing/scientific/FIT2D/

S4. H. Miura, *J. Crystallogr. Soc. Jpn.*, 2003, **45**, 145.

7. Supplementary tables

Table S1. XRD data for a film sample of TPC_4F_4* at 25 °C upon cooling from its isotropic liquid phase

Before shearing (D = 0 mm)				After shearing (D = 2.0 mm)			
q (nm^{-1})	$d_{obs.}$ (nm)	$d_{calc.}$ (nm)	*hkl*	q (nm^{-1})	$d_{obs.}$ (nm)	$d_{calc.}$ (nm)	*hkl*
2.69	2.34	2.33	100	5.32	1.18	1.17	200
4.69	1.34	1.34	110	8.03	0.78	0.78	300
5.42	1.16	1.17	200	10.78	0.58	0.59	400
7.10	0.89	0.81	210	10.99	0.57	0.55	211
8.06	0.78	0.77	300	12.25	0.51	0.51	410
9.31	0.68	0.67	220	13.00	0.48	0.48	311
9.69	0.65	0.65	310	17.80	0.35	0.35	002
10.76	0.58	0.58	400				
11.80	0.53	0.53	320				
12.36	0.51	0.51	410				

* *P*6*mm* hexagonal cell parameters at 25 °C for a film sample of **TPC_4F_4** before shearing: a = 2.69 nm, and that after shearing: a = 2.71 nm and c = 0.70 nm.

Table S2. XRD data for a film sample of TPC_4F_6*at 25 °C upon cooling from its isotropic liquid phase

Before shearing (D = 0 mm)				After shearing (D = 2.0 mm)			
q (nm^{-1})	$d_{obs.}$ (nm)	$d_{calc.}$ (nm)	*hkl*	q (nm^{-1})	$d_{obs.}$ (nm)	$d_{calc.}$ (nm)	*hkl*
2.45	2.56	2.36	100	2.40	2.62	2.36	100
4.28	1.47	1.36	110	4.83	1.30	1.18	200
4.98	1.26	1.18	200	6.28	1.00	0.89	210
6.02	1.04	0.89	210	8.05	0.78	0.79	300
7.97	0.79	0.79	300	11.22	0.56	0.55	211
11.95	0.53	0.54	320	12.08	0.52	0.54	320
				12.82	0.49	0.48	311
				17.95	0.35	0.35	002

* *P*6*mm* hexagonal cell parameters at 25 °C for a film sample of **TPC_4F_6** before shearing: a = 2.73 nm, and that after shearing: a = 2.73 nm and c = 0.70 nm.

Table S3. XRD data for a film sample of TPC_6F_4* at 25 °C upon cooling from its isotropic liquid phase

Before shearing (D = 0 mm)				After shearing (D = 2.0 mm)			
q (nm^{-1})	$d_{obs.}$ (nm)	$d_{calc.}$ (nm)	*hkl*	q (nm^{-1})	$d_{obs.}$ (nm)	$d_{calc.}$ (nm)	*hkl*
2.31	2.72	2.72	100	4.53	1.39		
4.01	1.57	1.57	110	5.22	1.20		
4.63	1.36	1.36	200	7.83	0.80		

* *P*6*mm* hexagonal cell parameter at 25 °C for a film sample of $\mathbf{TPC_6F_4}$ before shearing: a = 3.14 nm.

8. Supplementary figures

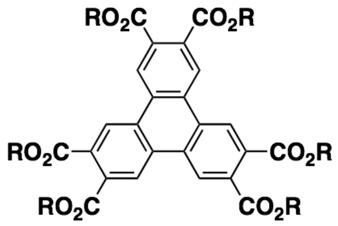

TPC$_2$: R = CH_2CH_3
TPC$_4$: R = $(CH_2)_3CH_3$
TPC$_6$: R = $(CH_2)_5CH_3$
TPC$_8$: R = $(CH_2)_7CH_3$
TPC$_{10}$: R = $(CH_2)_9CH_3$

Fig. S1. Molecular structures of $\mathbf{TPC_n}$ (ref. S2).

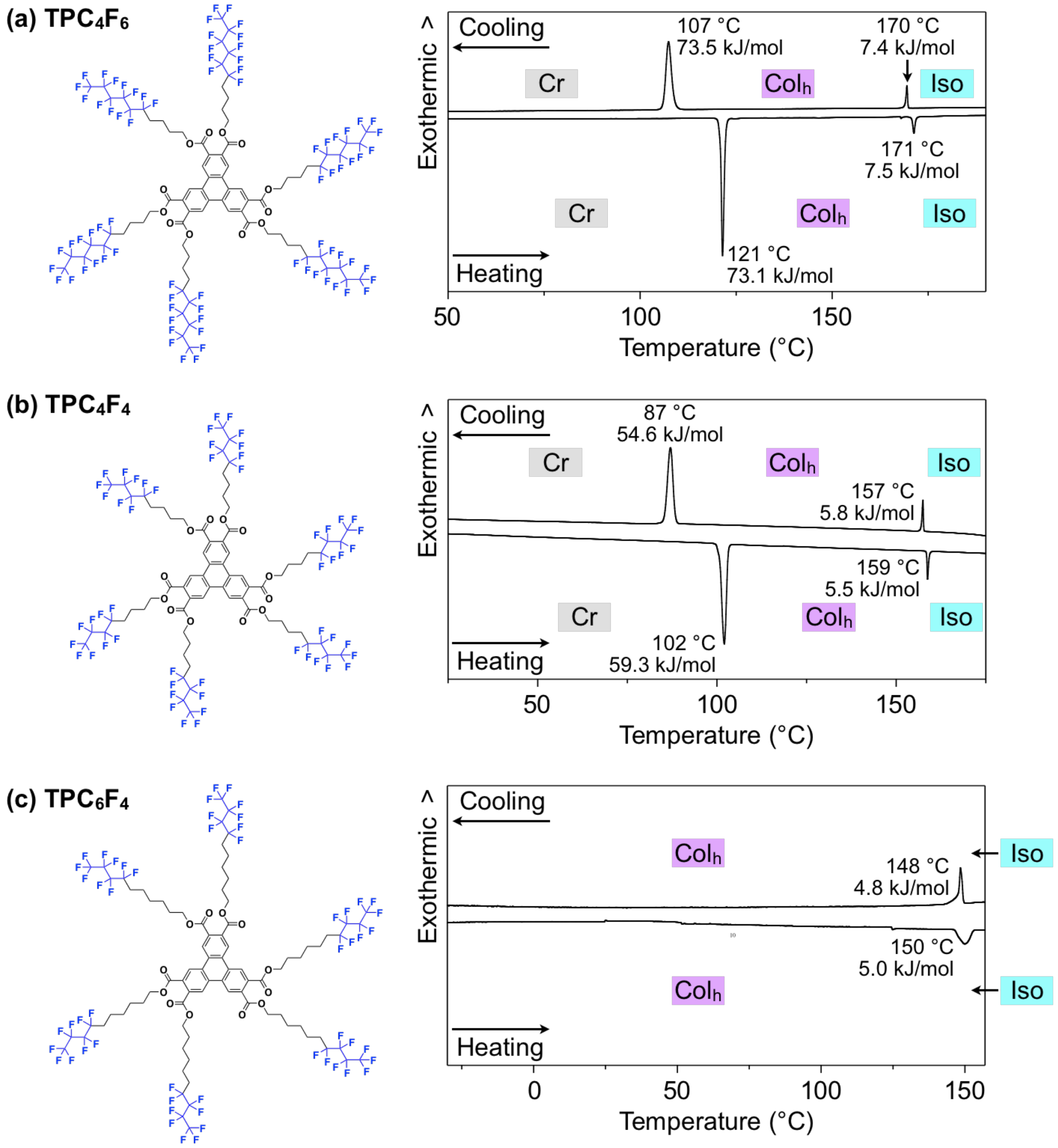

Fig. S2. Differential scanning calorimetry (DSC) profiles of (a) **TPC$_4$F$_6$**, (b) **TPC$_4$F$_4$**, and (c) **TPC$_6$F$_4$** measured at a scan rate of 5.0 °C/min (second heating/cooling cycle); Cr: crystal, Col$_h$: hexagonal columnar LC phase, Iso: isotropic liquid.

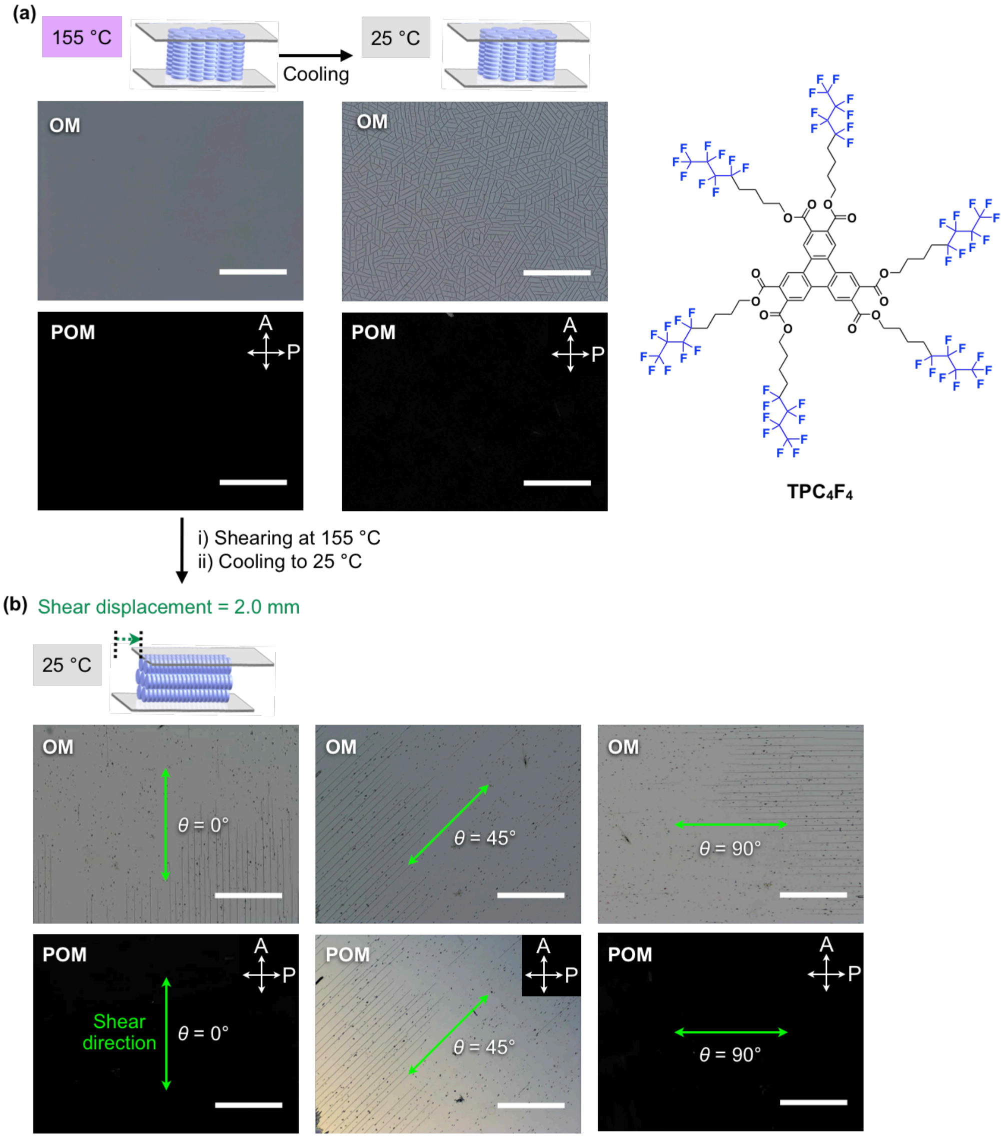

Fig. S3. (a) POM and OM images at 155 (left) and 25 °C (right) of an 8 μm-thick film of **TPC$_4$F$_4$** sandwiched between sapphire substrates. The film was once heated to the melting point of **TPC$_4$F$_4$** and subsequently cooled to the corresponding temperatures. (b) POM and OM images at 25 °C of an 8 μm-thick film of **TPC$_4$F$_4$** after mechanical shearing. The film was once heated to the melting point of **TPC$_4$F$_4$**, cooled to 155 °C, applied a shear force (shear displacement = 2.0 mm.) at the same temperature, and then cooled to 25 °C. White arrows represent the transmission axes of the polarizer (P) and analyzer (A), and green arrows and θ represent the shear direction and angles relative to the transmission axis of the analyzer, respectively. Cooling rate: 0.5 °C/min for the temperature range of the LC mesophase and >30 °C/min for the temperature range of the crystal phase. Scale bars = 200 μm.

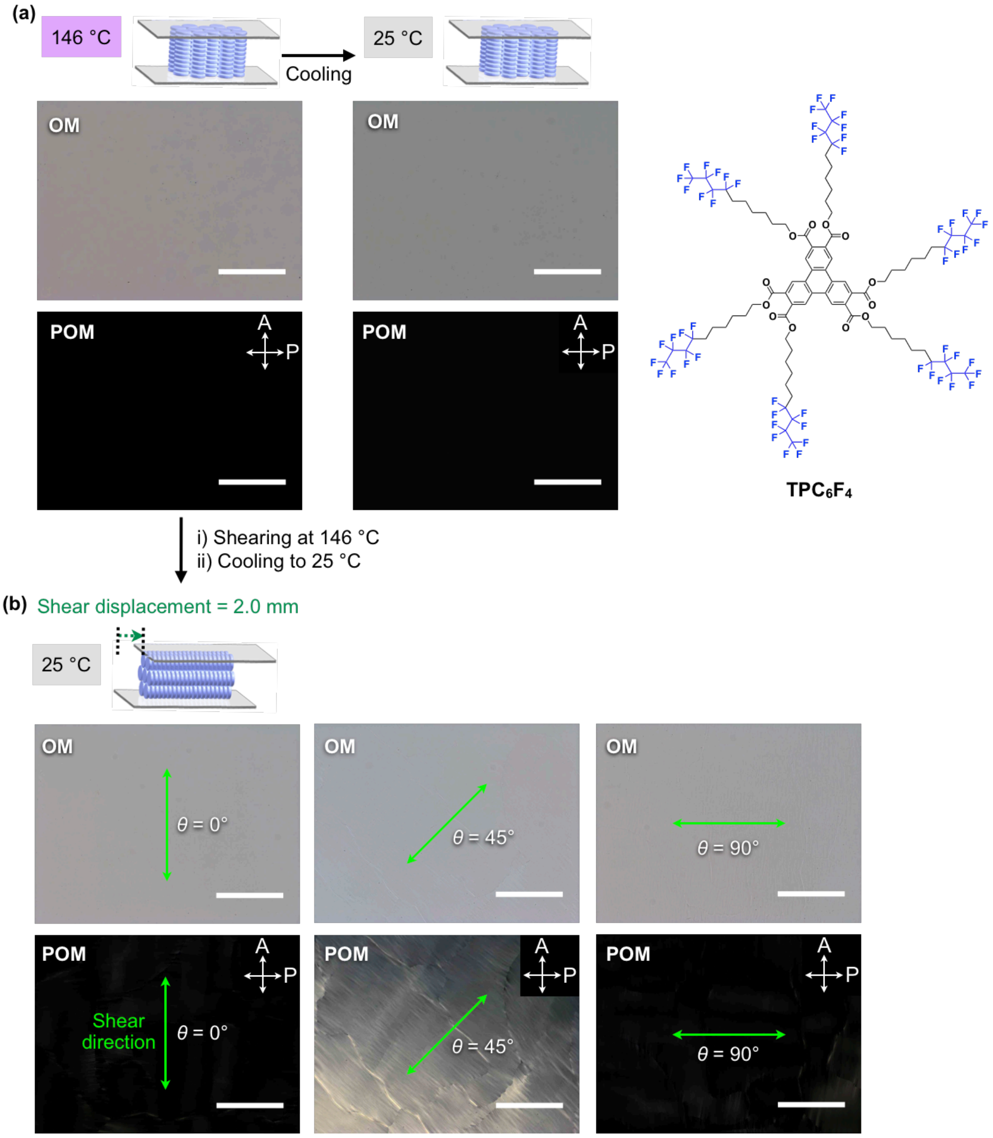

Fig. S4. (a) POM and OM images at 146 (left) and 25 °C (right) of an 8 μm-thick film of **TPC_6F_4** sandwiched between sapphire substrates. The film was once heated to the melting point of **TPC_6F_4** and subsequently cooled to the corresponding temperatures. (b) POM and OM images at 25 °C of an 8 μm-thick film of **TPC_6F_4** after mechanical shearing. The film was once heated to the melting point of **TPC_6F_4**, cooled to 146 °C, applied a shear force (shear displacement = 2.0 mm.) at the same temperature, and then cooled to 25 °C. White arrows represent the transmission axes of the polarizer (P) and analyzer (A), and green arrows and θ represent the shear direction and angles relative to the transmission axis of the analyzer, respectively. Cooling rate: 0.5 °C/min for the temperature range of the LC mesophase and >30 °C/min for the temperature range of the crystal phase. Scale bars = 200 μm.

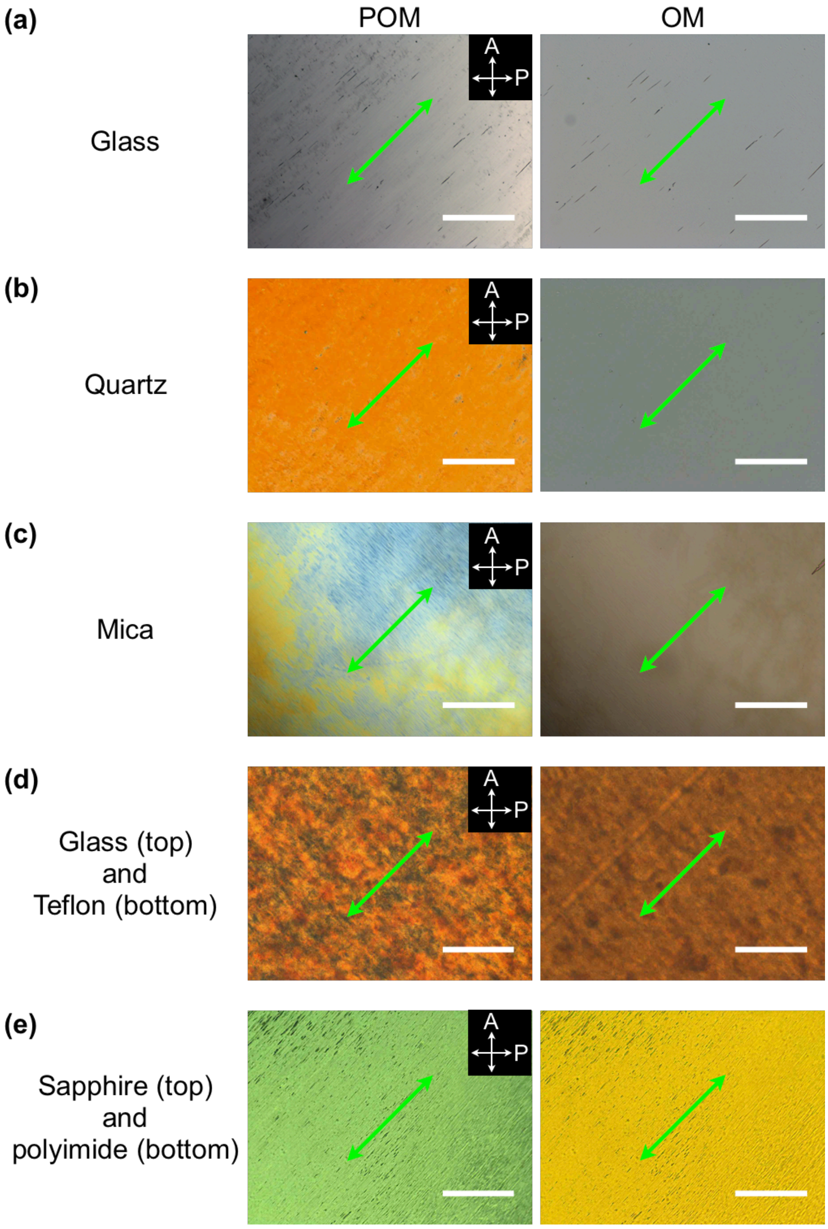

Fig. S5. POM (left) and OM (right) at 168 °C images of 8 μm-thick films of **TPC_4F_6** sandwiched between substrates after a shear-force treatment. Substrates: (a) glass/glass, (b) quartz/quartz, (c) mica/mica, (d) glass (top)/Teflon (bottom), and (e) sapphire (top)/polyimide (bottom). Each film was once heated to the melting point of **TPC_4F_6**, cooled to 168 °C (cooling rate: 0.5 °C/min), and then applied a shear force (shear displacement = 2.0 mm.) at 168 °C. The sample was tilted at 45° relative to the transmission axis of the polarizer. White arrows represent the transmission axes of the polarizer (P) and analyzer (A), and green arrows represent the shear direction. Scale bars = 200 μm.

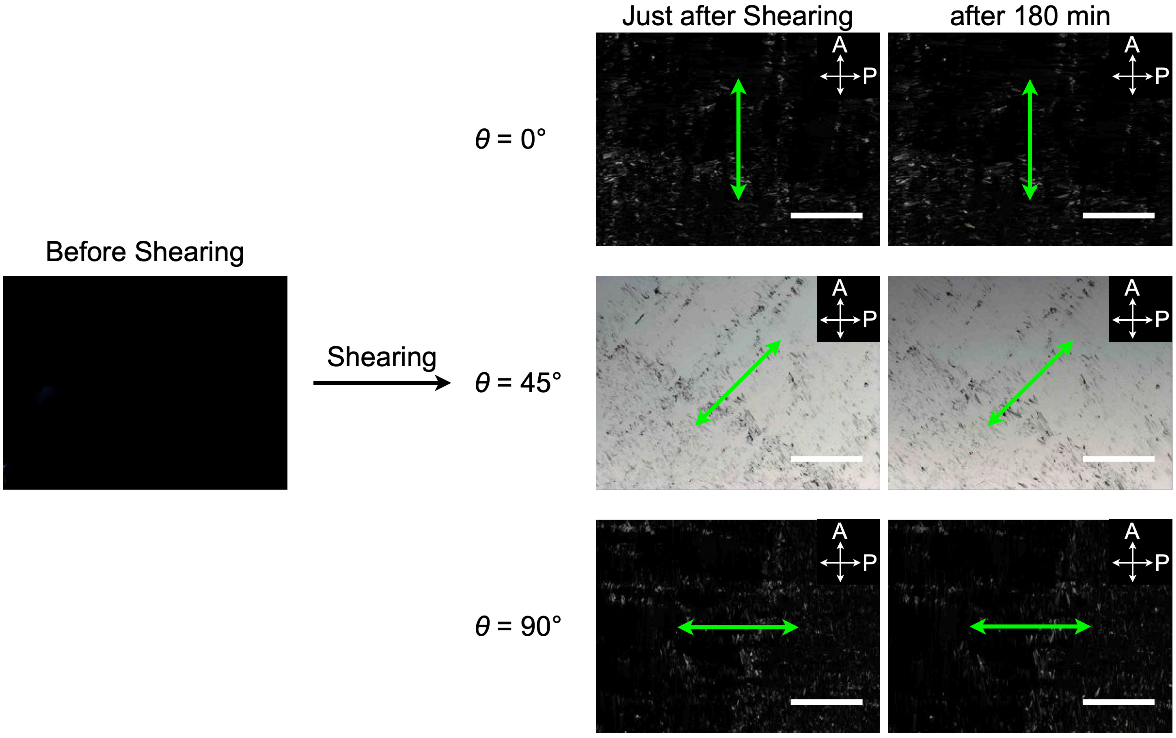

Fig. S6. POM images at 25 °C of a 20 μm-thick film of **TPC_4F_6** before and after mechanical shearing. The film was sandwiched between sapphire substrates, heated once to the melting point of **TPC_4F_6**, cooled to 160 °C, applied a shear force (shear displacement = 2.0 mm) at the same temperature, and then cooled to 25 °C. The sample was tilted at 0, 45, and 90° relative to the transmission axis of the analyzer. White arrows represent the transmission axes of the polarizer (P) and analyzer (A), and green arrows represent the shear direction. Cooling rate: 0.5 °C/min for the temperature range of the LC mesophase and >30 °C/min for the temperature range of the crystal phase. Scale bars = 200 μm.

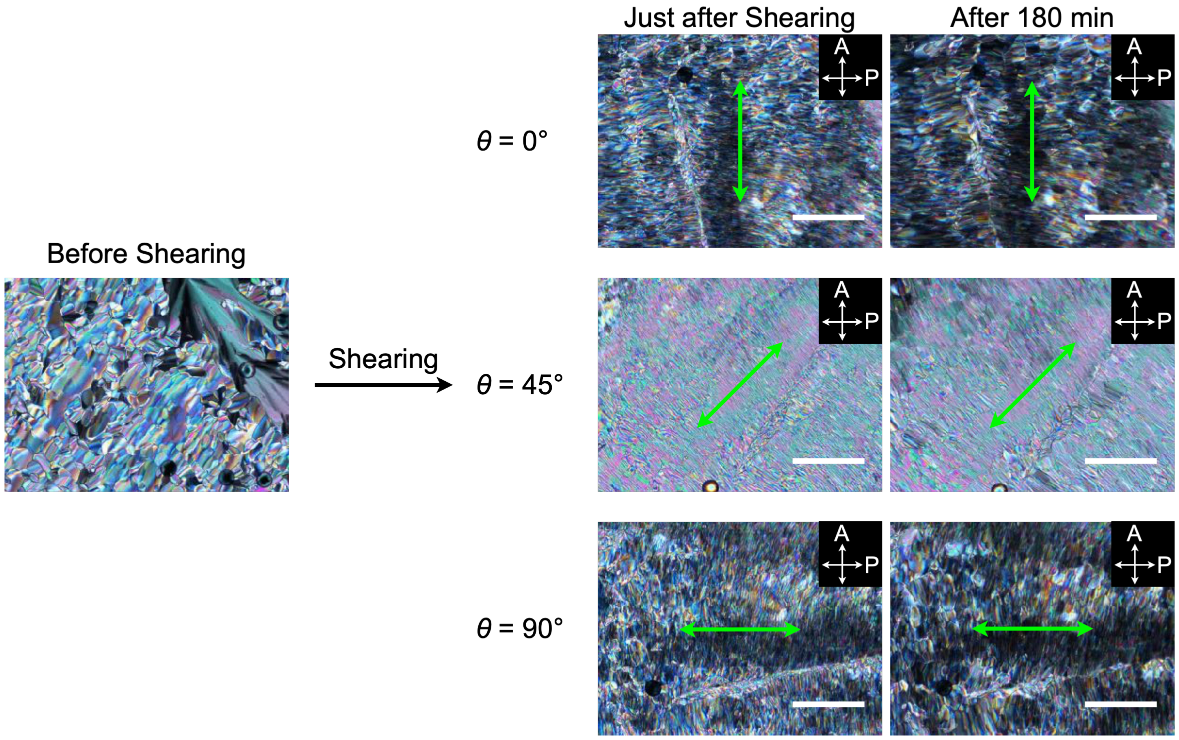

Fig. S7. POM images at 25 °C of a 50 μm-thick film of **TPC_4F_6** at 25 °C before and after mechanical shearing. The film was sandwiched between sapphire substrates, heated once to the melting point of **TPC_4F_6**, cooled to 160 °C, applied a shear force (shear displacement = 2.0 mm) at the same temperature, and then cooled to 25 °C. The sample was tilted at 0, 45, and 90° relative to the transmission axis of the analyzer. White arrows represent the transmission axes of the polarizer (P) and analyzer (A), and green arrows represent the shear direction. Cooling rate: 0.5 °C/min for the temperature range of the LC mesophase and >30 °C/min for the temperature range of the crystal phase. Scale bars = 200 μm.

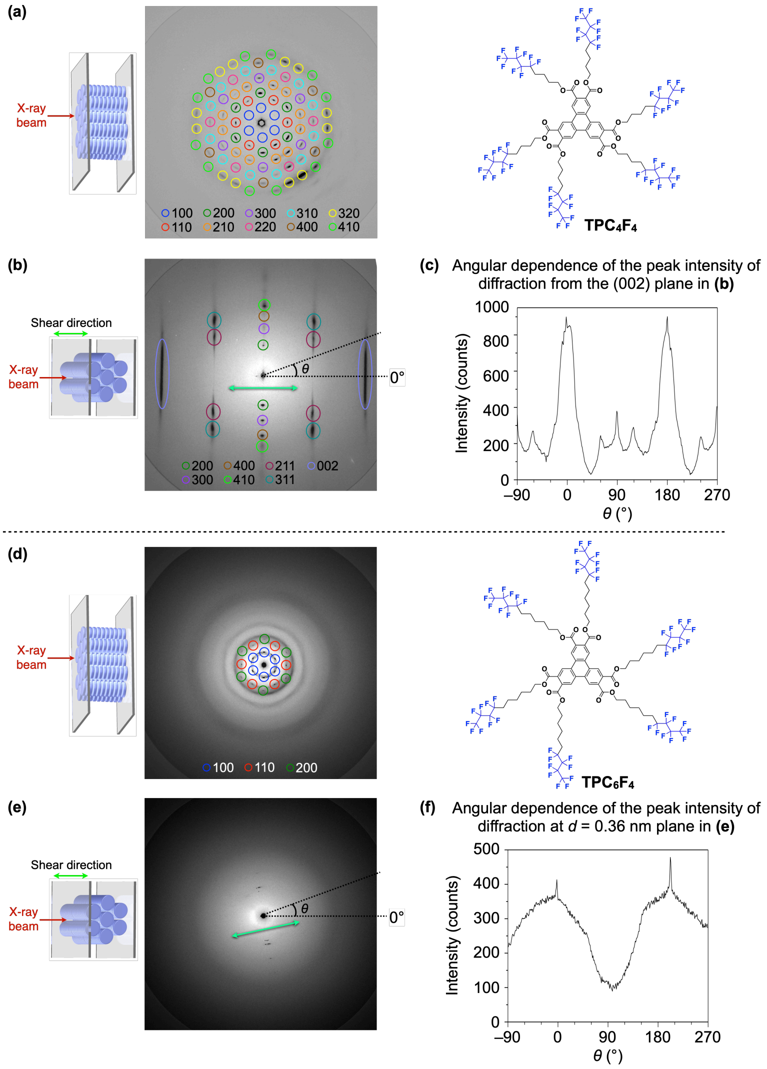

Fig. S8. Through-view two-dimensional (2D) XRD images at 25 °C of 8 μm-thick films of **TPC$_4$F$_4$** sandwiched between sapphire substrates, (a) prepared by cooling from its melting point to 25 °C and (b) prepared by cooling from its melting point to 155 °C, sheared at the same temperature (shear displacement = 2.0 mm, shear rate = 1.5 μm sec^{-1}), and then cooled to 25 °C. Cooling rate: 0.5 °C/min for the temperature range of the LC mesophase and >30 °C/min for the temperature range of the crystal phase. (c) Angular (θ) dependence of the peak intensity of diffraction from the (002) plane, obtained by converting the 2D XRD image in (b). Through-view two-dimensional (2D) XRD images at 25 °C of 8 μm-thick films of **TPC$_6$F$_4$** sandwiched between sapphire substrates, (d) prepared by cooling from its melting point to 25 °C and (e) prepared by cooling from its melting point to 146 °C, sheared at the same temperature (shear displacement = 2.0 mm, shear rate = 1.5 μm sec^{-1}), and then cooled to 25 °C. Cooling rate: 0.5 °C/min for the temperature range of the LC mesophase. (f) Angular (θ) dependence of the peak intensity of diffraction at d = 0.36 nm, obtained by converting the 2D XRD image in (e). Green arrows represent the shear direction.

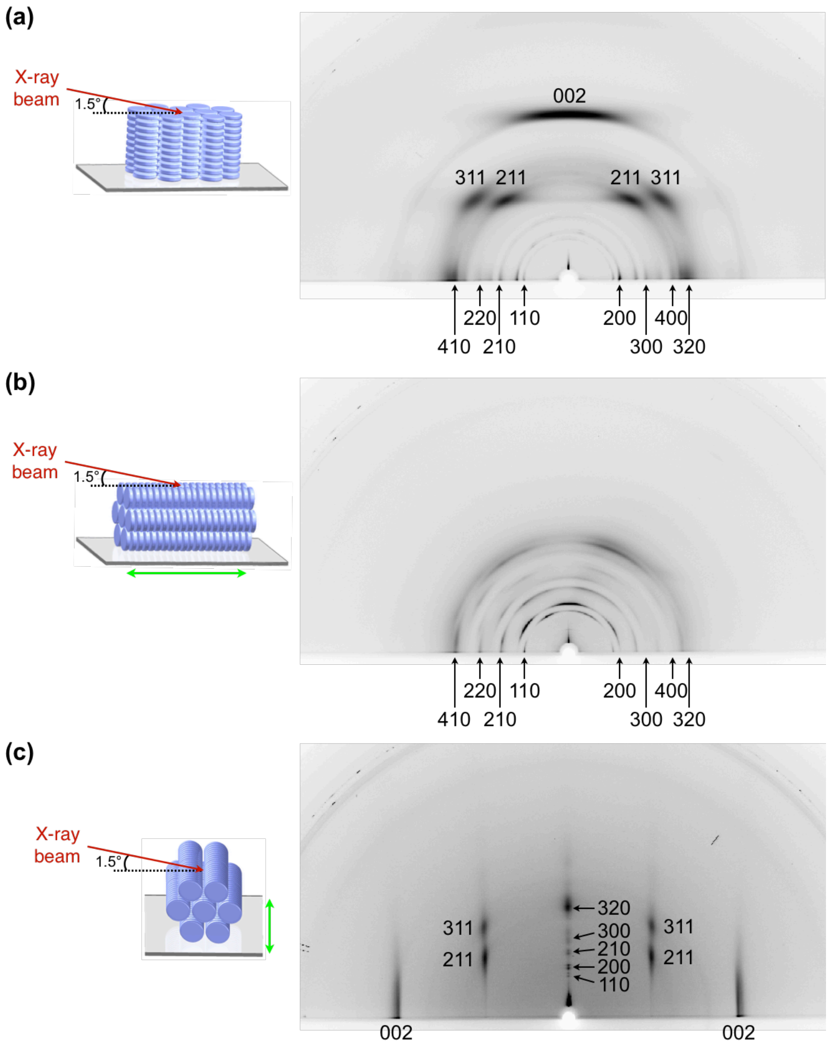

Fig. S9. 2D GI-XRD images at 25 °C of 8 μm-thick films of **TPC$_4$F$_4$** on a sapphire substrate, measured after removing the upper sapphire substrate from the corresponding sandwiched films. The sample for (a) was prepared by cooling from the melting point of **TPC$_4$F$_4$** to 25 °C. The samples for (b,c) were prepared by cooling from the melting point of **TPC$_4$F$_4$** to 155 °C, sheared at the same temperature (shear displacement = 2.0 mm, shear rate = 1.5 μm sec^{-1}), and then cooled to 25 °C. Cooling rate: 0.5 °C/min for the temperature range of the LC mesophase and >30 °C/min for the temperature range of the crystal phase. The directions of an incident X-ray beam for (b) and (c) were parallel and perpendicular to the shear direction, respectively, where the incident angle of the X-ray beam was 0.15°.

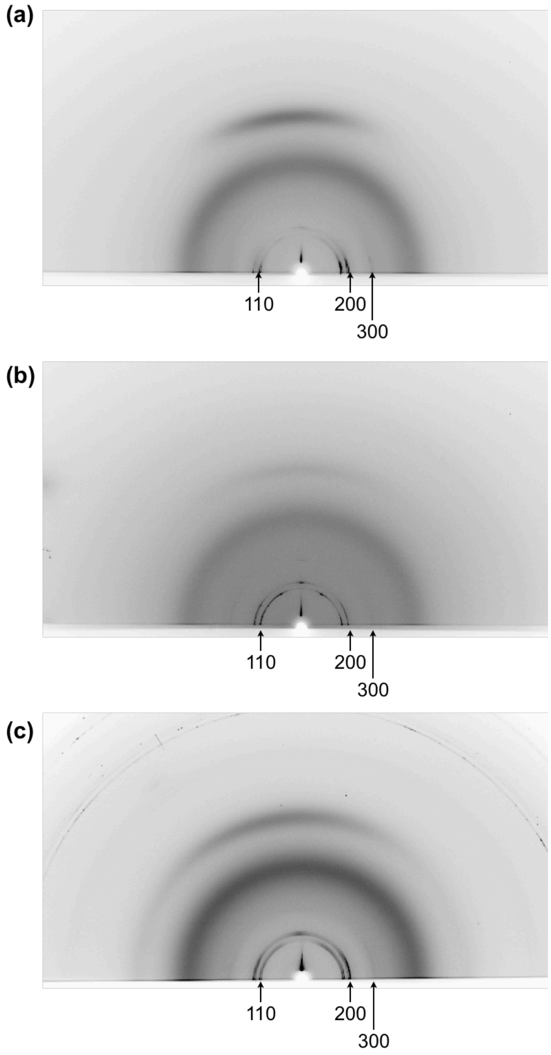

Fig. S10. 2D GI-XRD images at 25 °C of 8 μm-thick films of **TPC_6F_4** on a sapphire substrate measured after removing the upper sapphire substrate from the corresponding sandwiched films. The sample for (a) was prepared by cooling from the melting point of **TPC_6F_4** to 25 °C. The samples for (b,c) were prepared by cooling from the melting point of **TPC_6F_4** to 146 °C, sheared at the same temperature (shear displacement = 2.0 mm, shear rate = 1.5 μm sec^{-1}), and then cooled to 25 °C. Cooling rate: 0.5 °C/min for the temperature range of the LC mesophase. The directions of an incident X-ray beam for (b) and (c) were parallel and perpendicular to the shear direction, respectively, where the incident angle of the X-ray beam was 0.15°.

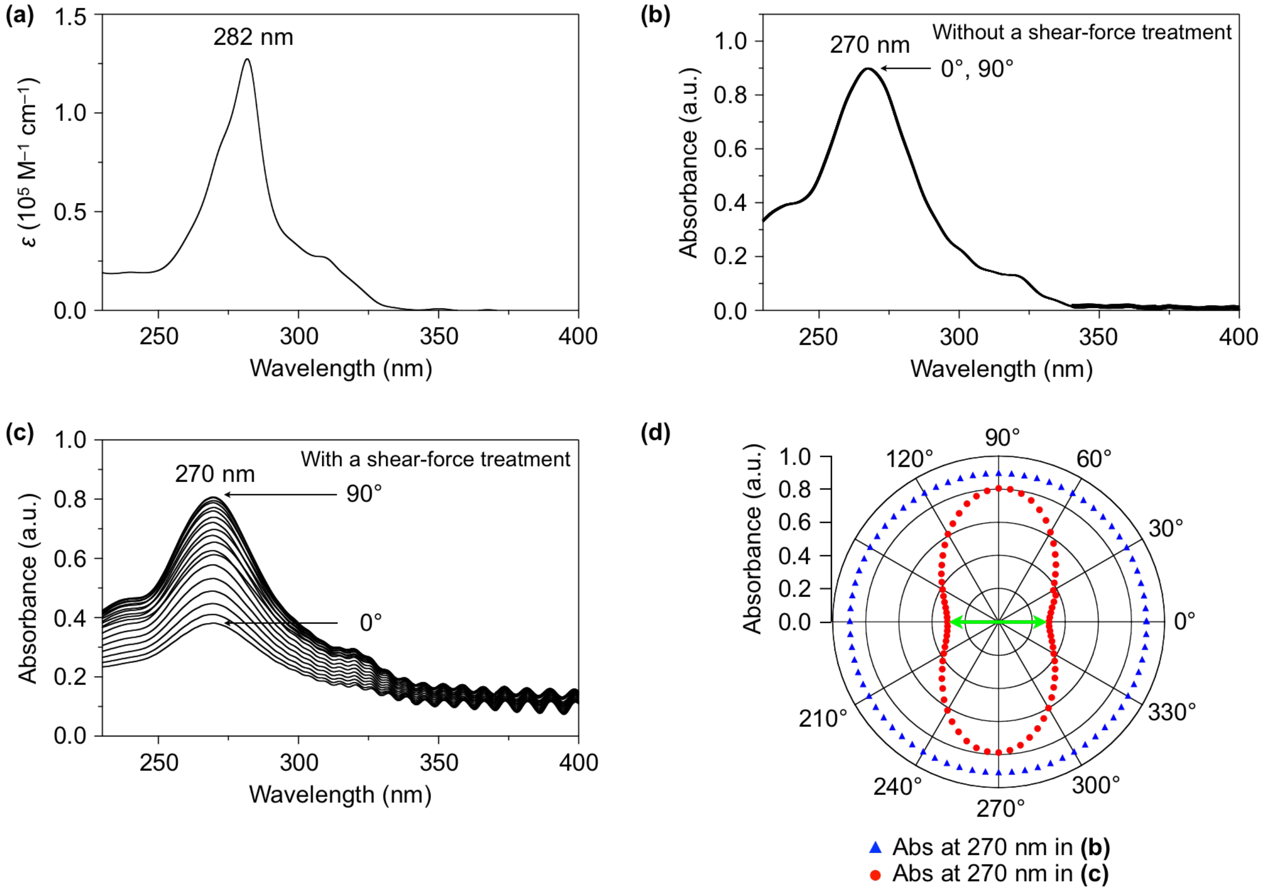

Fig. S11. (a) Electronic absorption spectrum of **TPC_4F_4** in CH_2Cl_2 (1.1 x 10^{-5} M) at 25 °C. (b,c) Polarized electronic absorption spectra (0° ≤ θ ≤ 90°) at 25 °C of an 8 μm-thick film of **TPC_4F_4** sandwiched between sapphire substrates, (b) prepared by cooling from the melting point of **TPC_4F_4** to 25 °C (cooling rate = 2.0 °C/min) and (c) prepared by cooling from the melting point of **TPC_4F_4** to 155 °C (cooling rate: 0.5 °C/min), sheared at the same temperature (shear displacement = 2.0 mm), and then cooled to 25 °C (cooling rate >30 °C/min). In (b) and (c), the wave-shaped feature observed above 300 nm is due to the light interference. The spectra were recorded while rotating the polarizer by 5°. (d) Polar plots obtained by converting the absorbance at 270 nm in (b) (blue) and (c) (red). The azimuthal angle (θ) is defined as zero when the polarizing direction of the incident light is parallel to the shear direction (green arrow).

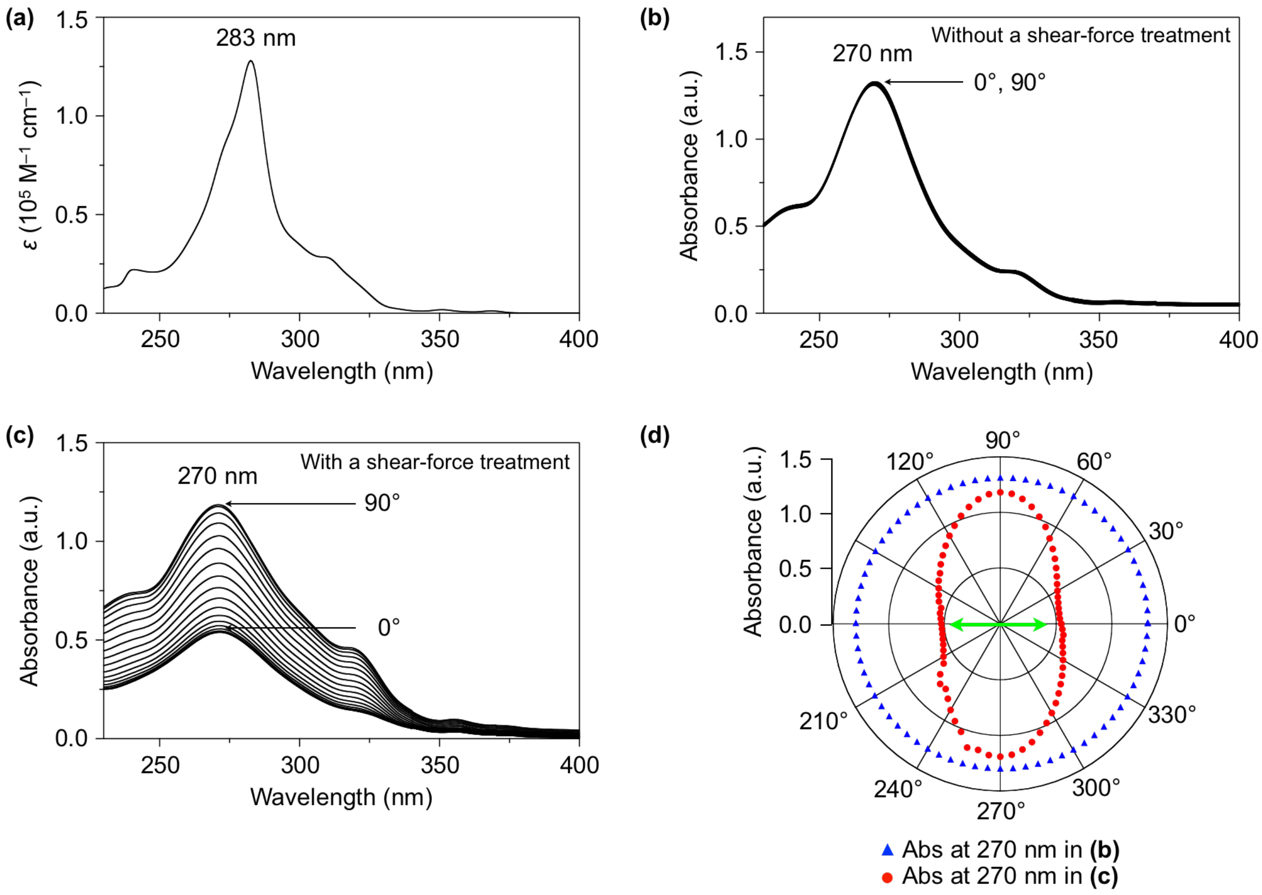

Fig. S12. (a) Electronic absorption spectrum of **TPC_4F_6** in CH_2Cl_2 (1.2 x 10^{-5} M) at 25 °C. (b,c) Polarized electronic absorption spectra (0° ≤ θ ≤ 90°) at 25 °C of an 8 μm-thick film of **TPC_4F_6** sandwiched between sapphire substrates, (b) prepared by cooling from the melting point of **TPC_4F_6** to 25 °C (cooling rate = 2.0 °C/min) and (c) prepared by cooling from the melting point of **TPC_4F_6** to 168 °C (cooling rate: 0.5 °C/min), sheared at the same temperature (shear displacement = 2.0 mm), and then cooled to 25 °C (cooling rate >30 °C/min). In (b) and (c), the wave-shaped feature observed above 300 nm is due to the light interference. The spectra were recorded while rotating the polarizer by 5°. (d) Polar plots obtained by converting the absorbance at 270 nm in (b) (blue) and (c) (red). The azimuthal angle (θ) is defined as zero when the polarizing direction of the incident light is parallel to the shear direction (green arrow).

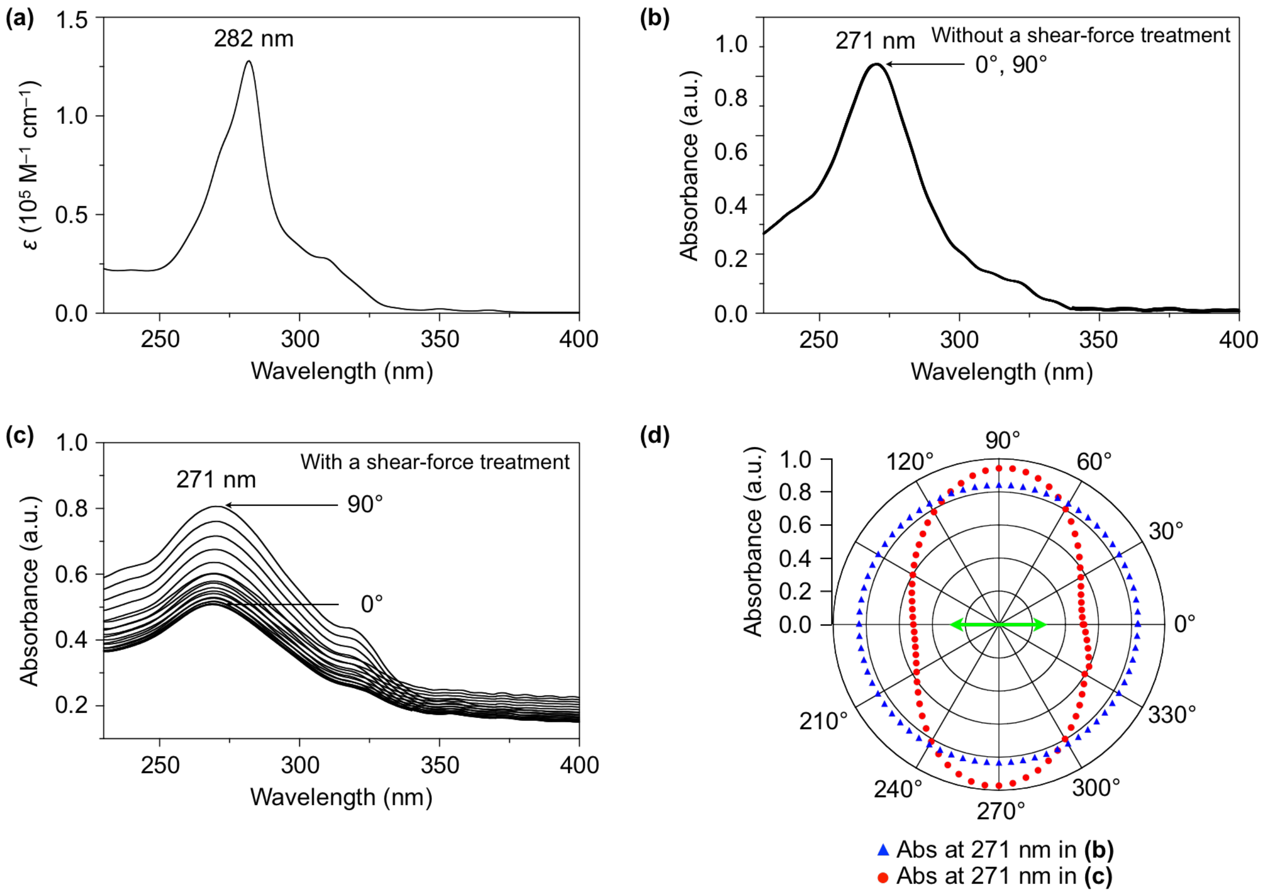

Fig. S13. (a) Electronic absorption spectrum of $\mathbf{TPC_6F_4}$ in CH_2Cl_2 (1.0×10^{-5} M) at 25 °C. (b,c) Polarized electronic absorption spectra ($0° \leq \theta \leq 90°$) at 25 °C of an 8 μm-thick film of $\mathbf{TPC_6F_4}$ sandwiched between sapphire substrates, (b) prepared by cooling from the melting point of $\mathbf{TPC_6F_4}$ to 25 °C (cooling rate = 2.0 °C/min) and (c) prepared by cooling from the melting point of $\mathbf{TPC_6F_4}$ to 146 °C (cooling rate: 0.5 °C/min), sheared at the same temperature (shear displacement = 2.0 mm), and then cooled to 25 °C (cooling rate >30 °C/min). In (b) and (c), the wave-shaped feature observed above 300 nm is due to the light interference. The spectra were recorded while rotating the polarizer by 5°. (d) Polar plots obtained by converting the absorbance at 271 nm in (b) (blue) and (c) (red). The azimuthal angle (θ) is defined as zero when the polarizing direction of the incident light is parallel to the shear direction (green arrow).

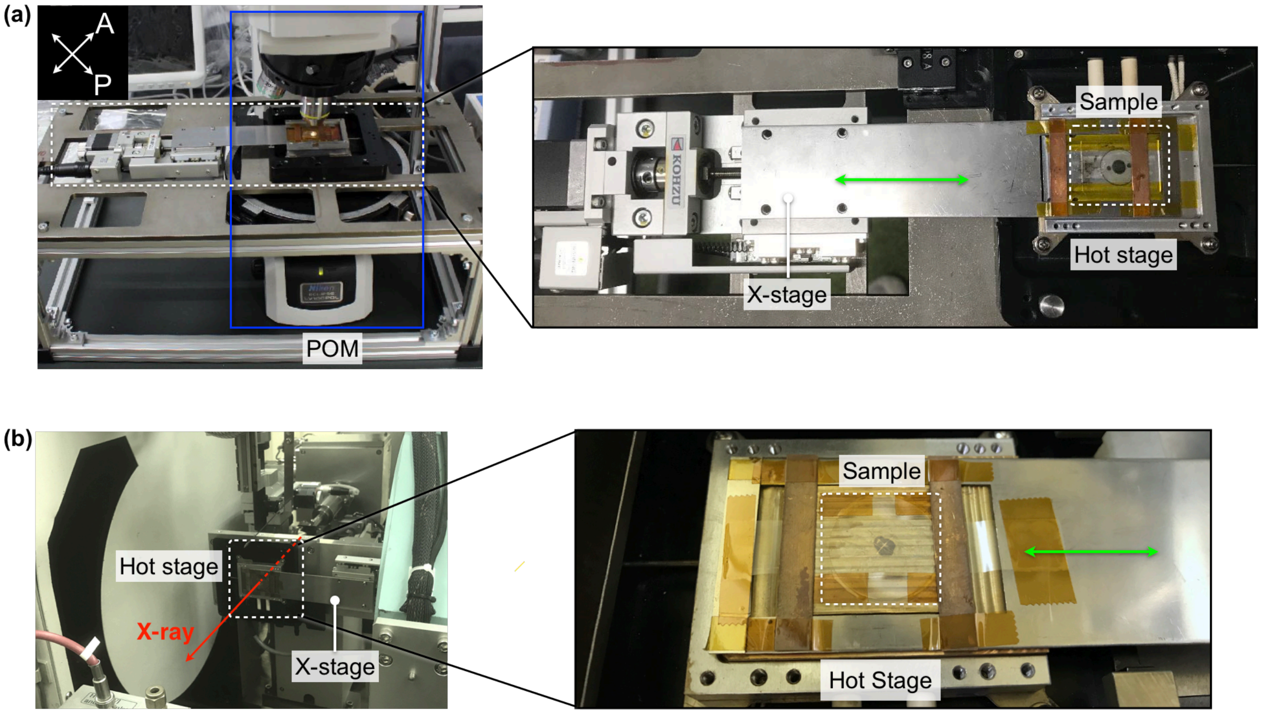

Fig. S14. Photographs of a dedicated experimental setup for (a) *in situ* POM measurements and (b) *in situ* through-view XRD measurements.

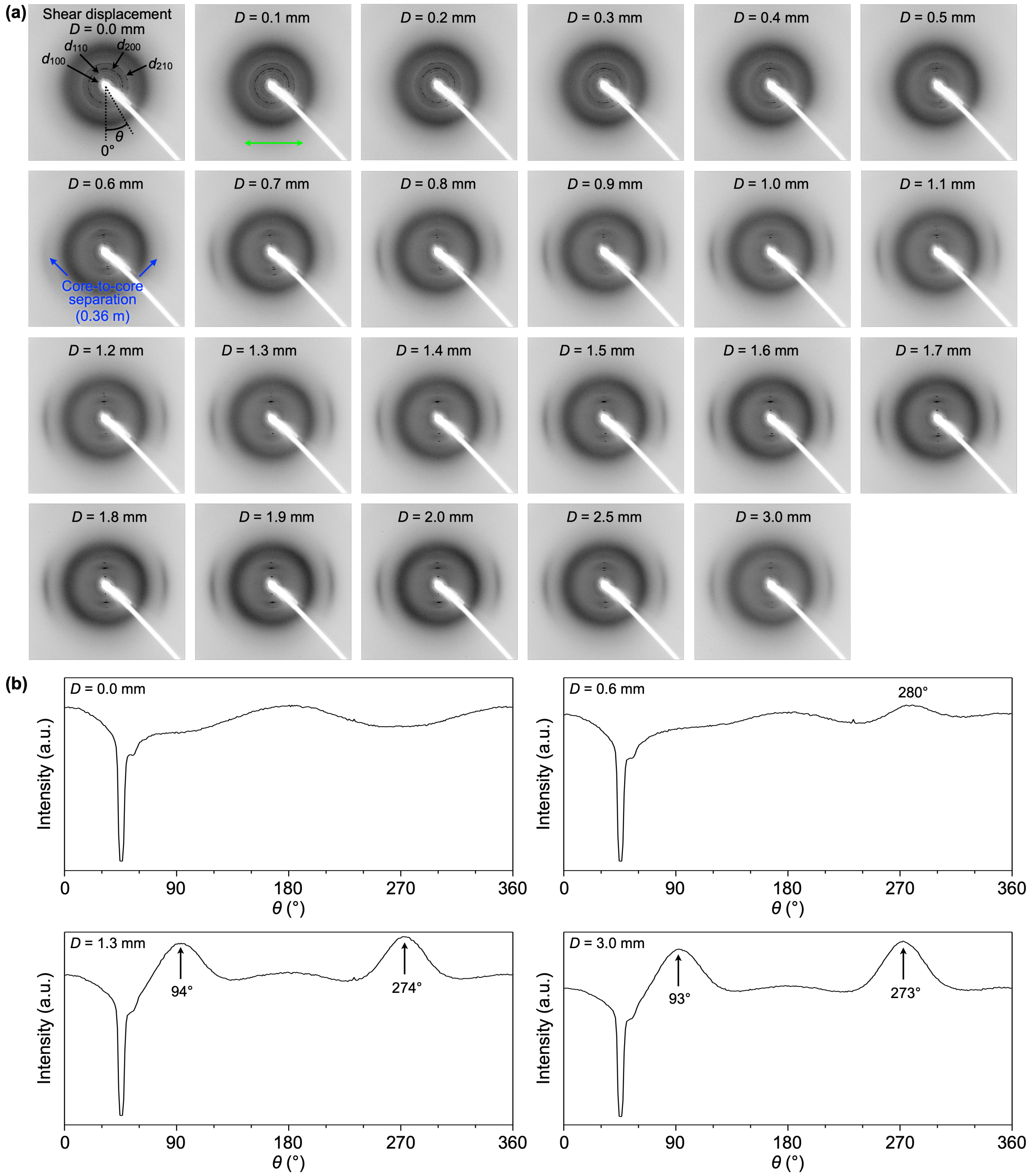

Fig. S15. (a) Full data sets for the *in situ* through-view XRD images of a 10 μm-thick film of **TPC_4F_6** at 168 °C under the application of a mechanical shear force (shear rate = 1.5 μm sec^{-1}) shown in Figure 6b. Photographs of the experimental setup are given in Figure S14b. The green arrow represents the shear direction. (b) Angular (θ) dependence of the peak intensity of diffraction from the (002) plane (*i.e.*, core-to-core separation) of the hexagonal columnar assembly, obtained by converting the 2D XRD images in (a).

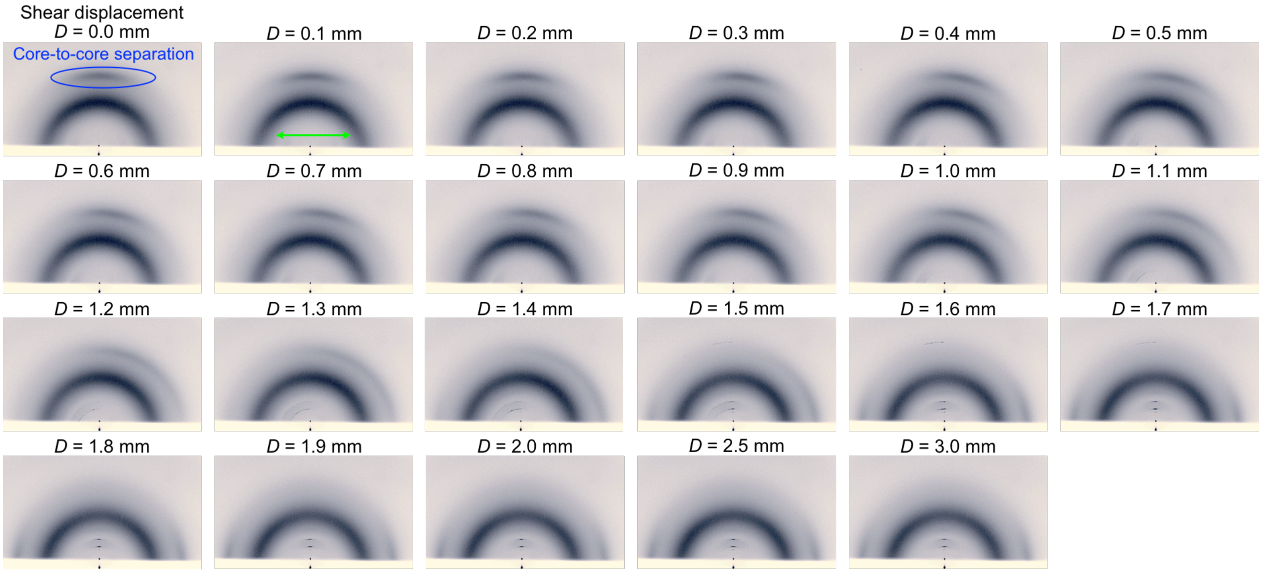

Fig. S16. Full data sets for the *in situ* reflective XRD images of a 10 μm-thick film of $\mathbf{TPC_4F_6}$ at 168 °C under the application of a mechanical shear force (shear rate = 1.5 μm sec^{-1}) shown in Figure 6d. Photographs of the experimental setup are given in Figure 6c. The green arrow represents the shear direction.

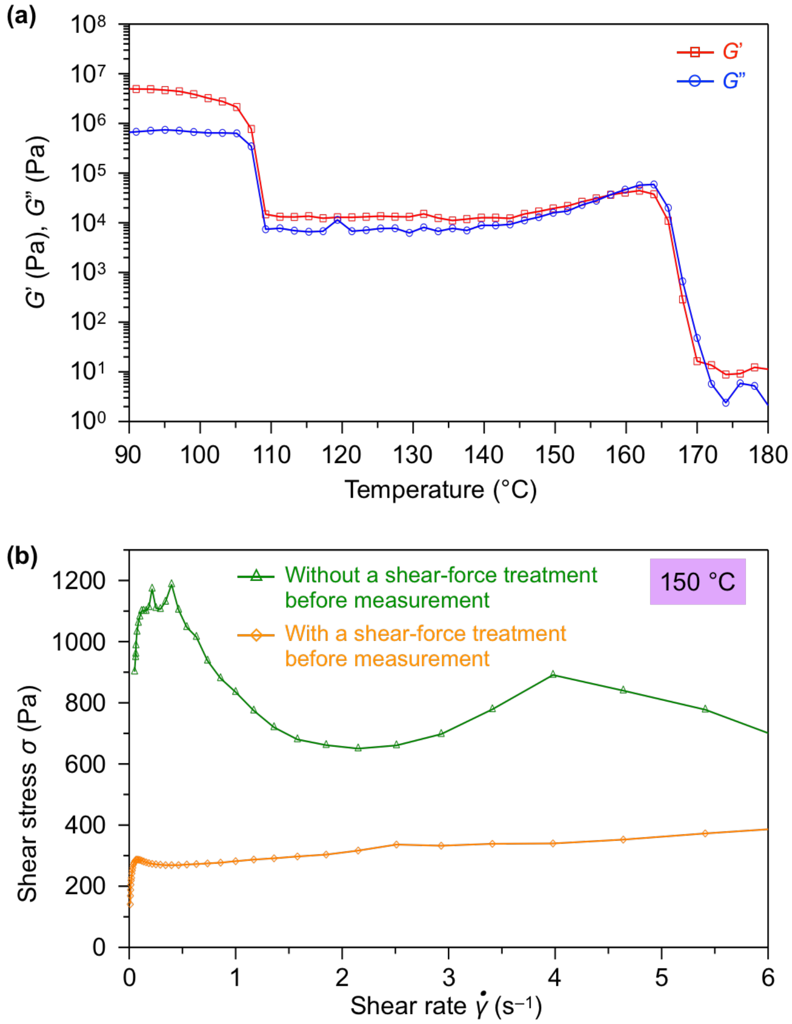

Fig. S17. Rheological properties of **TPC$_4$F$_4$**. (a) Temperature-dependence (in a heating process) of the storage modulus (*G*'; red) and loss modulus (*G*"; blue) measured under an applied strain (γ) of 0.1% and an angular frequency (ω) of 1.0 Hz. (b) Shear rate-dependence of shear stress at 150 °C without (green) and with (orange) shear-force treatment before measurement.
